# Runx2 Regulated Airway Homeostasis Is Disrupted in Asthma

**DOI:** 10.1096/fj.202502088R

**Published:** 2026-02-17

**Authors:** Junfei Wang, Alen Faiz, Qi Ge, Rob van de Velde, Theo Borghuis, Brian G. Oliver, Maarten van den Berge, Victor Guryev, Alan James, John G. Elliot, Andrew J. Halayko, Liang Dong, Anthony W. Ashton, Janette K. Burgess

**Affiliations:** ^1^ Department of Pulmonary and Critical Care Medicine Qilu Hospital of Shandong University Jinan Shandong China; ^2^ Woolcock Institute of Medical Research University of Sydney Sydney New South Wales Australia; ^3^ Department of Pulmonary Diseases University of Groningen, University Medical Center Groningen Groningen the Netherlands; ^4^ Faculty of Science, School of Life Sciences, Respiratory Bioinformatics and Molecular Biology (RBMB) University of Technology Sydney Ultimo New South Wales Australia; ^5^ GRIAC (Groningen Research Institute for Asthma and COPD) University of Groningen, University Medical Center Groningen Groningen the Netherlands; ^6^ Department of Pathology & Medical Biology University of Groningen, University Medical Center Groningen Groningen the Netherlands; ^7^ School of Life Sciences University of Technology Sydney Sydney New South Wales Australia; ^8^ Discipline of Pharmacology The University of Sydney Sydney New South Wales Australia; ^9^ University of Groningen, European Research Institute for the Biology of Ageing, Groningen University Medical Centre Groningen Groningen the Netherlands; ^10^ Department of Pulmonary Physiology and Sleep Medicine Sir Charles Gairdner Hospital Nedlands Western Australia Australia; ^11^ School of Medicine and Pharmacology The University of Western Australia Crawley Western Australia Australia; ^12^ University of Manitoba and Children's Hospital Research Institute of Manitoba Winnipeg Manitoba Canada; ^13^ Department of Respiratory and Intensive Care Unit The First Affiliated Hospital of Shandong First Medical University and Shandong Provincial Qianfoshan Hospital, Shandong Institute of Respiratory Diseases, Shandong Characteristic Laboratory of Clinical Transformation of Respiratory Biological Immunity and Regenerative Medicine Jinan Shandong China; ^14^ Division of Perinatal Research Kolling Institute of Medical Research Sydney New South Wales Australia; ^15^ Division of Cardiovascular Medicine Lankenau Institute for Medical Research Wynnewood Pennsylvania USA

**Keywords:** airway remodeling, airway smooth muscle, asthma, Runx2, Runx2 variants

## Abstract

In asthma, augmented airway wall smooth muscle (ASM) bulk is a major remodeling feature, promoted by increased transforming growth factor (TGF)‐β1 and connective tissue growth factor (CTGF). Runt‐related transcription factor‐2 (RUNX2) represses TGF‐β1‐induced CTGF through interactions with SMAD3. This study aimed to investigate the expression and role of RUNX2 in asthmatic and nonasthmatic ASM cells. mRNA and protein were detected by microarray, PCR, and western blot in nonasthmatic and asthmatic ASM cells. Immunohistochemistry identified RUNX2 in lung tissues from asthmatic patients and nonasthmatic subjects. Different RUNX2 isoforms were transfected into immortalized‐asthmatic ASM cells, and markers of inflammation and airway remodeling were measured. RUNX2 alternatively spliced forms were examined in bronchial biopsies from asthmatic and healthy subjects. The abundance of RUNX2 was decreased in isolated ASM cells from asthmatic compared with nonasthmatic subjects. The ASM layer around airways in lung tissue sections from asthmatic and nonasthmatic patients had a heterogeneous pattern of RUNX2 protein detection. TGF‐β1 stimulation increased RUNX2/RUNX2 variant 1 mRNA in nonasthmatic but not asthmatic ASM cells, facilitating SMAD3 activation and nuclear translocation in asthmatic ASM cells. RUNX2 isoform overexpression in immortalized asthmatic ASM cells failed to alter markers of inflammation (IL‐6) but significantly reduced markers of remodeling (CTGF), ASM cell hypertrophy (GSK‐3β and desmin), and proliferation (pSer^795^ Rb and α‐tubulin). In bronchial biopsies, RUNX2 mRNA splicing was higher in asthmatic patients compared with healthy subjects. These data suggest RUNX2 plays a role in the homeostasis of healthy airways. Restoring RUNX2 may provide a new therapeutic approach for asthma.

## Graphical abstract

Runt‐related transcription factor‐2 (RUNX2) expression and regulation of airway remodeling factors were examined in asthmatic and nonasthmatic airway smooth muscle (ASM) cells. Regulation by transforming growth factor‐β of active RUNX2 isoform translocation to the nucleus was absent in asthmatic ASM, while RUNX2 overexpression suppressed markers of remodeling in asthmatic ASM. Dysregulated RUNX2 in asthmatic ASM may contribute to disruption of airway homeostasis in asthma.

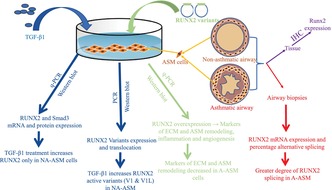

## 1 Introduction

Asthma is a complex multifactorial disease characterized by inflammation and structural changes in the airways [1]. The structural changes, termed airway remodeling, which include increased airway smooth muscle (ASM) bulk, subepithelial and ASM‐associated extracellular matrix (ECM) alterations, neovascularization, and mucus hypersecretion [2, 3, 4, 5], are linked to variable and excessive narrowing of the lumen and increased asthma severity [3, 6]. Current asthma therapies, including inhaled corticosteroids (ICS) and long‐acting beta2‐agonists (LABA), are effective in managing symptoms for most patients, but do not appear to affect airway remodeling [7, 8]. This highlights the urgent need for further understanding of the underlying mechanisms.

Increased ASM bulk within the airway wall has an important role in asthma pathophysiology. Apart from its structural role, causing reversible and excessive airway narrowing, ASM is also a potent source of cytokines, chemokines, and ECM proteins [9, 10, 11]. Transforming growth factor‐β1 (TGF‐β1), a governor of airway remodeling, is increased in the lungs of asthmatic subjects at both the gene and protein level [12, 13]. Many of the effects of TGF‐β1 are mediated through connective tissue growth factor (CTGF), the release of which is enhanced from asthmatic ASM [14, 15, 16, 17].

One of the dominant TGF‐β1 regulatory elements is the Runt‐related transcription factor (RUNX) family. RUNX2, also called CBFA1/OSF2/AML3/PEBP2aA [18], is a crucial transcription factor for bone formation, osteoblast differentiation, and maturation [19]. In addition to osteogenesis, RUNX2 was reported to be involved in cancer progression and tumorigenesis through modulating angiogenesis, cancer metastasis, proliferation, and drug resistance [20]. The RUNX2 gene, which is located in 6p21.1 in humans, encodes 12 transcript variants [20]. Expression of RUNX2 is regulated by two alternative promoters [21], which couple with alternative splicing of exon 7 to produce RUNX2 isoforms of varying biological function [22, 23]. RUNX2 isoforms containing exon 7 are able to translocate to the nucleus and repress SMAD3‐mediated TGF‐β1‐induced gene transcription in other systems [23, 24, 25], including the repression of CTGF expression, while isoforms lacking exon 7 remain in the cytoplasm. One study has shown that RUNX2 could promote asthma development by regulating goblet cell differentiation [26].

However, the expression and biological activity of RUNX2 have not been investigated in asthma to date. This study examined the expression of RUNX2 proteins in tissues from asthmatic patients (cell lines and airway tissues), and the functional role played by differentially expressed RUNX2 proteins in regulating airway remodeling.

## 2 Materials and Methods

### 2.1 In Silico Analysis of the CTGF Gene Promoter

Regulatory elements present in the lung‐specific regulatory region of the *CTGF* promoter were identified by scanning the region from −5452 to +200 bp of the *CTGF* gene (relative to the transcriptional start site) using the TFSiteScan database (http://www.ifti.org/cgi‐bin/ifti/Tfsitescan.pl).

### 2.2 Primary ASM Cell Isolation and Culture

Primary human ASM cells were isolated from explanted lung tissue or endobronchial biopsies from donors who provided written informed consent. Protocols were approved by the Ethics Review Committees of Royal Prince Alfred Hospital, the South West Sydney Area Health Service, Strathfield Private Hospital, St Vincent's Hospital Sydney, and the University of Sydney Human Research Ethics Committee. Clinical characteristics of the donors are in Table 1. In vitro cultures of primary ASM cells were established and maintained as previously [9, 27], in 10% (v/v) fetal bovine serum (FBS) in high glucose (4.5 g/L) Dulbecco's modified Eagle's medium (DMEM, Sigma‐Aldrich, St. Louis, MO, USA) with 100 U/mL penicillin, 100 μg/mL streptomycin, and 0.25 μg/mL Amphotericin B (Thermofisher, Waltham, MA USA). All primary ASM cells were used for experimentation between Passages 2 and 8.

**TABLE 1:** Details of asthmatic and nonasthmatic subjects used for establishment of airway smooth muscle cells.

Patients	Age	Sex	Diagnosis	Sample type
1	20	Male	Asthmatic	Biopsy
2	62	Male	Cancer	Resection
3	45	Male	Asthmatic	Biopsy
4	23	Male	Asthmatic	Biopsy
5	27	Female	Asthmatic	Biopsy
6	57	Male	Cancer	Resection
7	22	Female	Healthy	Biopsy
8	62	Male	Cancer	Resection
9	67	Male	Healthy	Transplant
10	61	Male	Cancer	Resection
11	38	Male	Asthmatic	Biopsy
12	70	Male	Cancer	Resection
13	62	Male	Cancer	Resection
14	43	Male	Healthy	Transplant
15	66	Male	Cancer	Resection
16	21	Male	Asthmatic	Biopsy
17	47	Male	Healthy	Transplant
18	68	Male	Cancer	Resection
19	78	Male	Cancer	Resection
20	52	Male	Cancer	Resection
21	29	Male	Normal	Biopsy
22	61	Female	Asthmatic	Biopsy
23	60	Female	Cancer	Resection
24	71	Female	Cancer	Resection
25	61	Male	Cancer	Resection
26	54	Male	Asthmatic	Biopsy
27	85	Male	Asthmatic	Biopsy
28	51	Male	Asthmatic	Biopsy

*Note:* All asthmatic subjects had a doctor's diagnosis of asthma.

### 2.3 ASM Cell Stimulation

Nonasthmatic (NA) and asthmatic (A) ASM cells were seeded in six‐well plates (10^4^ cells/cm^2^), in 5% (v/v) FBS/DMEM and grown for 3 days before quiescing for 24 or 72 h in 0.1% (w/v) BSA/DMEM followed by stimulation with recombinant human TGF‐β1 (1 ng/mL, R&D Systems, Minneapolis, USA).

### 2.4 Transfection of RUNX2 Variants Into ASM Cells

Human TERT‐immortalized A‐ASM cell lines (iA‐ASM) [28] were transfected with ORF constructs (2 μg/10^6^ cells) for *RUNX2* isoforms ([V1: Genescript (Piscataway, NJ)] [V1l, V2: BIOMATIC (Wilmington, DE)]) using the Amaxa Basic Nucleofector Kit (VPI‐1004, Lonza). The base vector (pcDNA3.1) was used as a negative control in all experiments. Cells were plated in six‐well plates (10^4^ cells/cm^2^) for 10 h with 10% (v/v) FBS/DMEM, quiesced in 0.1% (w/v) BSA for 14 h, and stimulated with TGF‐β1 (1 ng/mL) for 2, 12, and 24 h. Supernatants were collected for ELISA analysis and the cell lysates for mRNA and protein detection.

### 2.5 RNA Extraction

Primary and transfected iA‐ASM cells were harvested in lysis buffer and total RNA isolated according to the manufacturer's instructions (ISOLATE RNA mini kit; Bioline, London, UK). RNA concentration was quantified with a Nanodrop 2000 Spectrophotometer (NanoDrop Technologies, Wilmington, DE, USA) before storage at −20°C.

### 2.6 Microarray Analysis

Human ASM cells were obtained from bronchial biopsies and explanted lungs from doctor diagnosed asthmatic patients (*n* = 3) and healthy controls (*n* = 3). ASM cells were isolated and grown in culture as previously described [29, 30]. Cells at passage 3–4 were seeded into six‐well plates and grown to confluence. Cells were then quiesed for 72 h and treated with/without TGF‐β1 (10 ng/mL) for 8 h. Total cellular mRNA was isolated using the Qiagen total RNA isolation kit (Qiagen, Doncaster, Victoria, Australia). Samples were labeled and run on Affymetrix (Santa Clara, California, USA) GeneChip Human Gene 1.0 ST Arrays (GSE63383) as described previously [29]. Microarray analysis was conducted using R software V.3.02, (Free Software Foundation Inc) using the Bioconductor‐limma package, and normalized using Robust Multi‐array Average.

### 2.7 Real‐Time Reverse Transcriptase Polymerase Chain Reaction (RT‐PCR)

Gene expression profiles were determined using the BioSense SensiFast Probe Hi‐ROX Mastermix (Bioline) in a StepOne Plus detection system (Thermo Fisher) with specific primers (Table 2). 18s rRNA was used for normalization and relative gene expression was calculated using the 2^−∆∆Ct^ method.

**TABLE 2:** Primers for detection of genes of interest from Life Technologies.

Primer name	Primer catalogue number
RUNX2	Hs00231692_m1
SMAD3	Hs00969210_m1
Interleukin 6 (IL‐6)	HS00174131‐m1
Fibronectin (*FN‐1*)	HS01549959‐m1
Vascular endothelial growth factor (*VEGF‐A*_*165*_)	HS 00173626‐m1
18s rRNA	4319413E‐1 011 052

### 2.8 Detection of RUNX2 Variants Using Reverse Transcriptase PCR

RNA from NA‐ and A‐ASM cells was analyzed using reverse transcriptase PCR with isoform specific primers (Table 3) using MyTaq HS Red DNA Polymerase (Bioline). PCR products were loaded in agarose gel and images were captured and analyzed by a Kodak Image station 4000 mm.

**TABLE 3:** Primer sequence for Runx2 variants expression.

Primer name	Primer sequence		Annealing temperature	Cycle #
Runx2 Variant1	Forward	CCTCAGTGATTTAGGGCGCA	56	30
Runx2 Variant2	Reverse	TGCCTGGGGTCTGAAAAAGG		
Runx2 P1	Forward	CAAACAGCCTCTTCAGCACAG	54	30
Runx2 P2 5'UTR	Forward	TCGCTAACTTGTGGCTGTTG	52	30
Runx2 P1 + P2	Reverse	GGCTCACGTCGCTCATTTT		
GAPDH	Forward	TCTAGACGGCAGGCTAGGTCCACC	60	25
	Reverse	CCACCCATGGCAAATTCCATGGCA		

### 2.9 Detection of RUNX2 by Immunohistochemistry

Immunohistochemistry staining for RUNX2 was performed on 4‐μm‐thick sections of formalin‐fixed paraffin‐embedded lung tissue from donors with asthma who died from asthma (fatal asthma) or other causes (nonfatal asthma) or without asthma. Sections were deparaffinized and antigen retrieval was performed in 0.1 M citrate pH 6.0 buffer, by incubating the sections for 15 min at 100°C in a microwave. Slides were incubated overnight at 4°C with primary antibody anti‐RUNX2 1:100 (ThermoFisher, PA5‐82787), followed by anti‐rabbit horseradish peroxidase‐conjugated secondary antibody 1:200 (DAKO, P0448). Staining was visualized using NovaRED (VECTOR laboratories, SK4805), and hematoxylin was used as a counterstain.

#### 2.9.1 Image Analysis

Tissue sections were scanned using a NanoZoomer XR digital slide scanner (Hamamatsu Photonics). FIJI ImageJ was used to quantify the density and distribution of the staining. Color deconvolution [31] vectors in FIJI were optimized to ensure accurate separation of hematoxylin and NovaRED. To remove tar staining in the lung tissue sections a third color vector was added. To measure the total tissue area a new image was created by combining the hematoxylin and NovaRED images and subtracting the tar images from the combined image using the image calculator in FIJI. The image analysis calculated the number of pixels above a certain threshold within the image (area of positive pixels) and the average intensity. Data of RUNX2 expression were represented as:

$$\left(\text{NovaRed area}/\text{Total tissue area}\right)\times 100.$$

### 2.10 Detection of RUNX2 and SMAD3 in ASM by Immunofluorescence

Immunofluorescent staining of NA‐ and A‐ASM (*n* = 5 each) for RUNX2 and SMAD3 was performed on paraformaldehyde fixed cells. After plating on coverslips, cells were stimulated with TGF‐β1 (1 ng/mL, 2 h), washed with PBS, fixed in 2% (w/v) paraformaldehyde and permeabilized with 1% (v/v) Triton X‐100 in PBS. Permeabilized cells were blocked for 1 h with 5% (w/v) BSA in PBST followed by anti‐RUNX2 or anti‐SMAD3 (1:1000 each) for 1 h. After washing, cells were incubated with the appropriate AlexaFluor 488 conjugated second antibody for 1 h, washed in PBS‐T and mounted in aqueous media containing DAPI (Vector Labs Inc). Cells were imaged with a Nikon ECLIPSE 80i fluorescent microscope with FITC filters, using a Nikon Digital Sight Control Unit, Nikon Digital Sight DS‐5 M camera and NIS‐Elements software version 3 for imaging (Nikon Australia, Sydney, NSW).

### 2.11 Cell Fractionation, Protein Analysis and Immunoblotting

For whole cell lysates, ASM cells were washed twice with ice cold PBS and lysed with RIPA buffer (20 mM Tris–HCl, pH 7.4, 150 mM NaCl, 1 mM Na_2_EDTA, 1 mM EGTA, 1 mM NaF, 20 mM Na_4_P_2_O_7_, 2 mM Na_3_VO_4_, 1% (v/v) Triton X‐100, 10% (v/v) glycerol, 0.1% (w/v) SDS, 0.5% (w/v) sodium deoxycholate, 1 mM phenylmethylsulfonyl fluoride (PMSF), and 1:100 protease inhibitor cocktail Set III (Merck‐Millipore, California, USA)). Lysates were incubated on ice for 30 min and sonicated briefly (5 s, 15% output) before being clarified by centrifugation (10 min, 16 000 × *g*). Supernatants were stored at −20°C until further analysis.

For fractionation into nuclear and cytoplasmic compartments, ASM cells were washed twice with ice‐cold PBS, scraped into PBS, and recovered by centrifugation at 15 000 × *g* for 30 s. The resultant pellet was re‐suspended in 300 μL of buffer A (10 mM Hepes, pH 7.91, 10 mM KCl, 0.1 mM EDTA, 0.1 mM EGTA, 1 mM DTT, 0.5 mM PMSF, 1 μg/mL pepstatin A, 0.1 mM benzamadine, 1 μg/mL leupeptin, 10 μg/mL aprotinin) by gentle pipetting and incubated on ice for 15 min. After the addition of 18.75 μL 10% (v/v) NP‐40, the mixture was vortexed for 10 s and centrifuged at 15 000 × *g* for 30 s. The supernatant, containing the cytoplasmic protein fraction, was collected and stored at −20°C for further analysis. The pellet was re‐suspended with 100 μL Buffer C (20 mM Hepes, pH 7.91, 400 mM KCl, 1 mM EDTA, 1 mM EGTA, 1 mM DTT, 1 mM PMSF, 1 μg/mL pepstatin A, 0.1 mM benzamadine, 1 μg/mL leupeptin, 10 μg/mL aprotinin) and incubated at 4°C for 15 min on a shaking platform before being centrifuged at 15 000 × *g* for 5 min. The supernatant containing the nuclear proteins was collected and stored as above.

Protein concentrations were quantified using a Pierce BCA Protein Assay Kit (Life Technologies). Samples were heated (95°C for 10 min) prior to separation by SDS‐PAGE gel to ensure denaturation of the contained proteins.

Protein extracts (50 μg/lane) were separated on a 10% (w/v) SDS‐PAGE gel and transferred to polyvinylidene difluoride (PVDF) membrane (Merck‐Millipore). Membranes were blocked in Tris buffered saline (TBS) with 0.05% (v/v) tween 20 (TBS‐T) containing 5% (w/v) skim milk at RT for 60 min before incubation with primary antibodies against RUNX2 (1:1000, ab23981, Abcam), SMAD3 (1:2000, ab28379, Abcam), total and pSer^795^ Rb (1:2000, 9969, Cell Signaling Technologies), desmin (sc‐23 879), total (sc‐7291) and p‐Ser^9^ (sc‐373 800) Glycogen synthase kinase 3β (GSK‐3β), Glyceraldehyde 3‐phosphate dehydrogenase (GAPDH) (sc‐47 724) (1:2000, Santa Cruz), or α‐tubulin (1:5000, T9026, Sigma) in TBS‐T with 5% (w/v) skim milk at 4°C overnight. After washing, the membranes were incubated with HRP‐coupled secondary antibody (1:5000 antirabbit (P0448) or 1:5000 for antimouse (P0160), DAKO). Expression was visualized using enhanced chemiluminescence (PerkinElmer) and an ImageQuant LAS 4000 imaging system (GE Healthcare). Densitometry of protein bands was quantified using FIJI Image J software [32, 33, 34].

### 2.12 ELISA

IL‐6 and VEGF‐A_165_ release were measured according to the manufacturer's instructions (R&D Systems, Minneapolis, Minnesota, USA).

### 2.13 RUNX2 Gene Expression and Correlation With Clinical Disease

#### 2.13.1 Bronchial Biopsy Processing for Quantification of RUNX2 Expression

Bronchial biopsies were collected from respiratory healthy subjects (*n* = 77) [35], asthmatic taking ICS (*n* = 44) and asthmatics not taking ICS (*n* = 25) [36, 37]. Biopsies were taken from segmental divisions of the main bronchi. All patients had a previous doctor's diagnosis of asthma, documented reversibility and AHR to histamine (PC20 histamine, using 30‐s tidal breathing, < 32 mg/mL). An outline of the patients' characteristics has been previously published [37, 38]. All study protocols were approved by the University Medical Center Groningen medical ethics committee and all subjects provided written informed consent.

#### 2.13.2 RNA Extraction, Sample Preparation, and High‐Throughput Sequencing

Biopsies frozen in Tissue‐Tek (VWR, Radnor, PA) at −80°C were thawed at RT and cut from the blocks when they were semi‐solid. Total RNA was extracted using AllPrep DNA/RNA Mini kit (Qiagen, Venlo, the Netherlands). Samples were lysed in 600 μL RLT‐plus buffer using an IKA Ultra Turrax T10 Homogenizer, and RNA was purified according to the manufacturer's instructions. RNA samples were dissolved in 30 μL RNAse free water. Concentrations and quality of RNA were checked using a Nanodrop‐1000 and run on a Labchip GX (PerkinElmer, Waltham, MA).

RNA samples were further processed using the TruSeq Stranded Total RNA Sample Preparation Kit (Illumina, San Diego, CA), using an automated procedure in a Caliper Sciclone NGS Workstation (PerkinElmer, Waltham, MA). In this procedure, all cytoplasmic and mitochondrial rRNA was removed (RiboZero Gold kit). The obtained cDNA fragment libraries were loaded in pools of multiple samples unto an Illumina HiSeq2500 sequencer using default parameters for paired‐end sequencing (2 × 100 bp).

#### 2.13.3 Gene Expression Quantification

The trimmed fastQ files where aligned to build b37 of the human reference genome using HISAT (version 0.1.5) allowing for 2 mismatches [39]. Before gene quantification SAMtools (version 1.2) was used to sort the aligned reads [40]. The gene level quantification was performed by HTSeq (version 0.6.1p1) using Ensembl version 75 as gene annotation database [41].

#### 2.13.4 Quality Control

Quality control (QC) metrics were calculated for the raw sequencing data, using the FastQC tool (version 0.11.3). Alignments of 220 subjects were obtained. QC metrics were calculated for the aligned reads using Picard‐tools (version 1.130) (http://picard.sourceforge.net) CollectRnaSeqMetrics, MarkDuplicates, CollectInsertSize‐Metrics and SAMtools flagstat. We discarded 36 samples due to poor alignment metrics. In addition, we checked for concordance between sex‐linked (*XIST* and Y‐chromosomal genes) gene expression and reported sex. All samples were concordant.

#### 2.13.5 Differential Expression

Raw counts of expressed features were analyzed using the R‐package DESeq2 [40]. Feature counts were set as the dependent variable, smoking status was investigated correcting for age and gender. The use of splice sites was quantified by counting split reads mapping across exon–exon junctions using a custom in‐house script (available upon request). Split reads in less than 5% of all individuals were removed from the analysis [36].

### 2.14 Statistical Analysis

Data analyses were performed using Microsoft Excel (Microsoft, Redmond, WA, USA) and statistical analyses performed and graphs produced in GraphPad Prism version 6 (GraphPad Software, La Jolla, CA, USA). Data were tested for normal distribution and analyzed via a two‐way analysis of variance (ANOVA) with Bonferroni post‐test. A *p* value less than 0.05 was considered significant (*p* < 0.05).

## 3 Results

### 3.1 RUNX Binding Is a Unique Feature of the Lung‐Specific Regulatory Region of the CTGF Promoter

**FIGURE 1:**
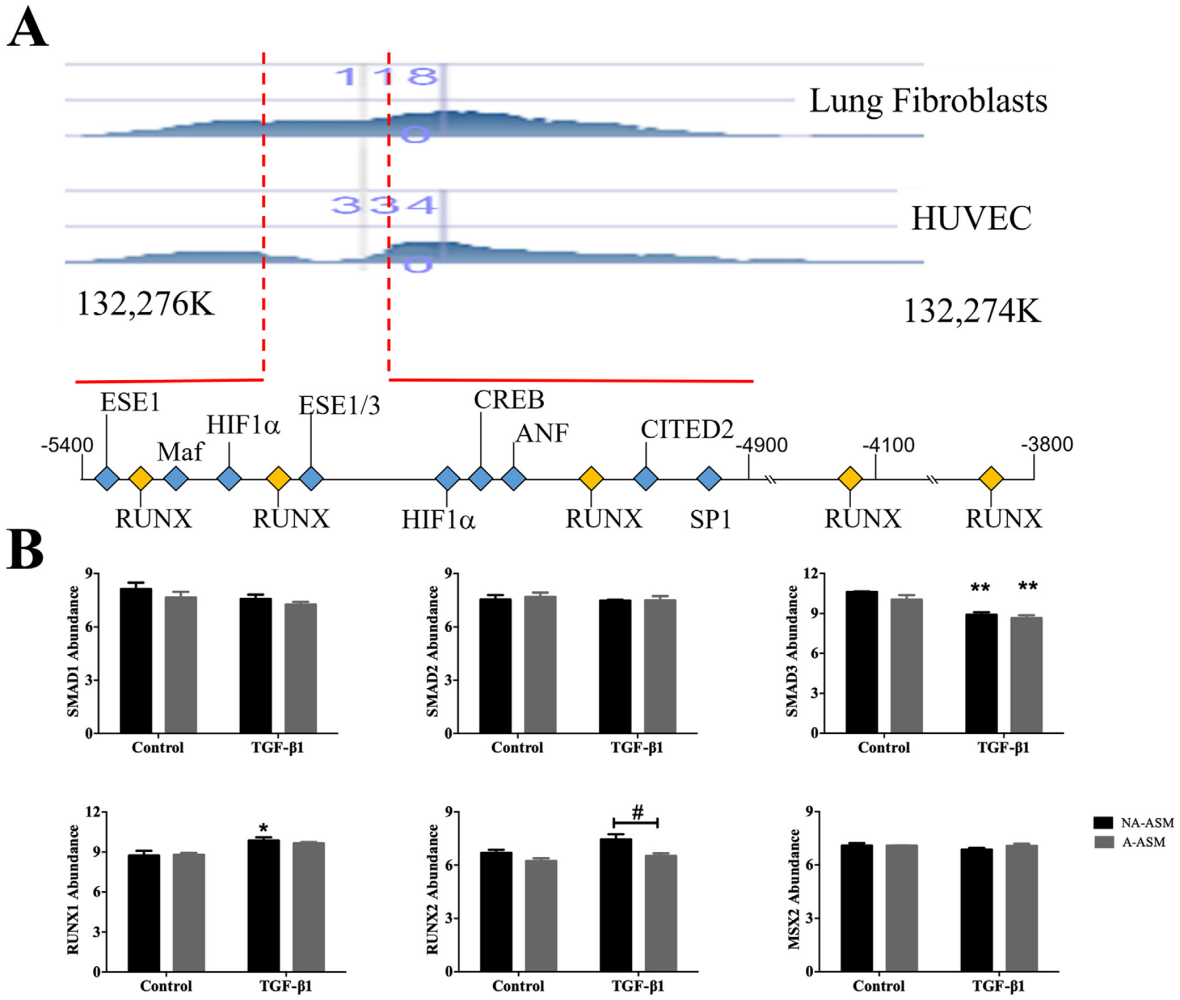
Activation of the upstream *CTGF* promoter may be due to RUNX2 dysregulation in A‐ASM cells. (A) In silico analysis of the upstream “promoter” (−5400 to −3400 bp) in the *CTGF* gene reveals a series of putative binding sites for transcription factors. Detailed analysis of the “lung specific” region identified consensus binding sites for RUNX proteins as points of difference. (B) Microarray expression profiling of downstream effectors of TGF‐β1 signaling in NA‐ (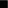) and A‐ASM (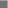) cells (*n* = 3 of each). Data represent fold change to untreated control (mean ± SEM). *p < 0.05, **p < 0.01 denotes significance between BSA and TGF‐β1. ^#^ *p* < 0.05 indicates significant difference between NA‐ and A‐ASM cells.

We have previously identified a novel, lung‐specific 5′ regulatory region in the TGF‐β1 responsive gene, *CTGF* [42]. Analysis of the 5′ regulatory region (−5400 to −3400) identified multiple putative transcription factor binding sites (Figure 1A, Table S1); however, multiple consensus binding sites for the RUNX family of transcriptional regulators were of interest given previous associations with TGF‐β1 signaling. Thus, we investigated whether any of the RUNX family were differentially expressed in asthmatic airways.

### 3.2 RUNX2 Abundance in Response to TGF‐β1 Is Decreased in ASM From Asthmatic Patients

Microarray analysis of the TGF‐β1 signaling pathway in NA‐ and A‐ASM cells found equivalent abundance of the effectors *SMAD1* and *2* and this did not change with TGF‐β1 stimulation, whilst *SMAD3* abundance decreased in both NA‐ (*n* = 3, *p* < 0.01) and A‐ASM (*n* = 3, *p* < 0.01) cells (Figure 1B). Abundance of *RUNX1* was TGF‐β1 inducible in NA‐ASM cells (*n* = 3, *p* < 0.05) but did not differ between cell types. In contrast to all other effectors assessed, *RUNX2* abundance was higher in NA‐ (*n* = 3) than A‐ASM cells (*n* = 3, *p* < 0.05) after TGF‐β1 treatment.

We also investigated binding partners for RUNX2. Abundance of the cognate RUNX2 inhibitor *MSX2* was similar in NA‐ and A‐ASM and unchanged by TGF‐β1 treatment (*n* = 3, Figure 1B). Indeed, when we examined all reported RUNX2 binding partners by microarray analysis (Table S2), the only changes observed were the reduced abundance of *UBTF* and *WWP1*. *UBTF* is a target of RUNX2‐mediated transcriptional repression through deacetylation by histone deacetylase (HDAC)1. *WWP1* encodes an E3‐ligase that mediates RUNX2 degradation. The simultaneous reduction of these proteins with RUNX2 is potentially a mechanism for restoring homeostasis by repressing pathways that become deregulated in the absence of RUNX2.

**FIGURE 2:**
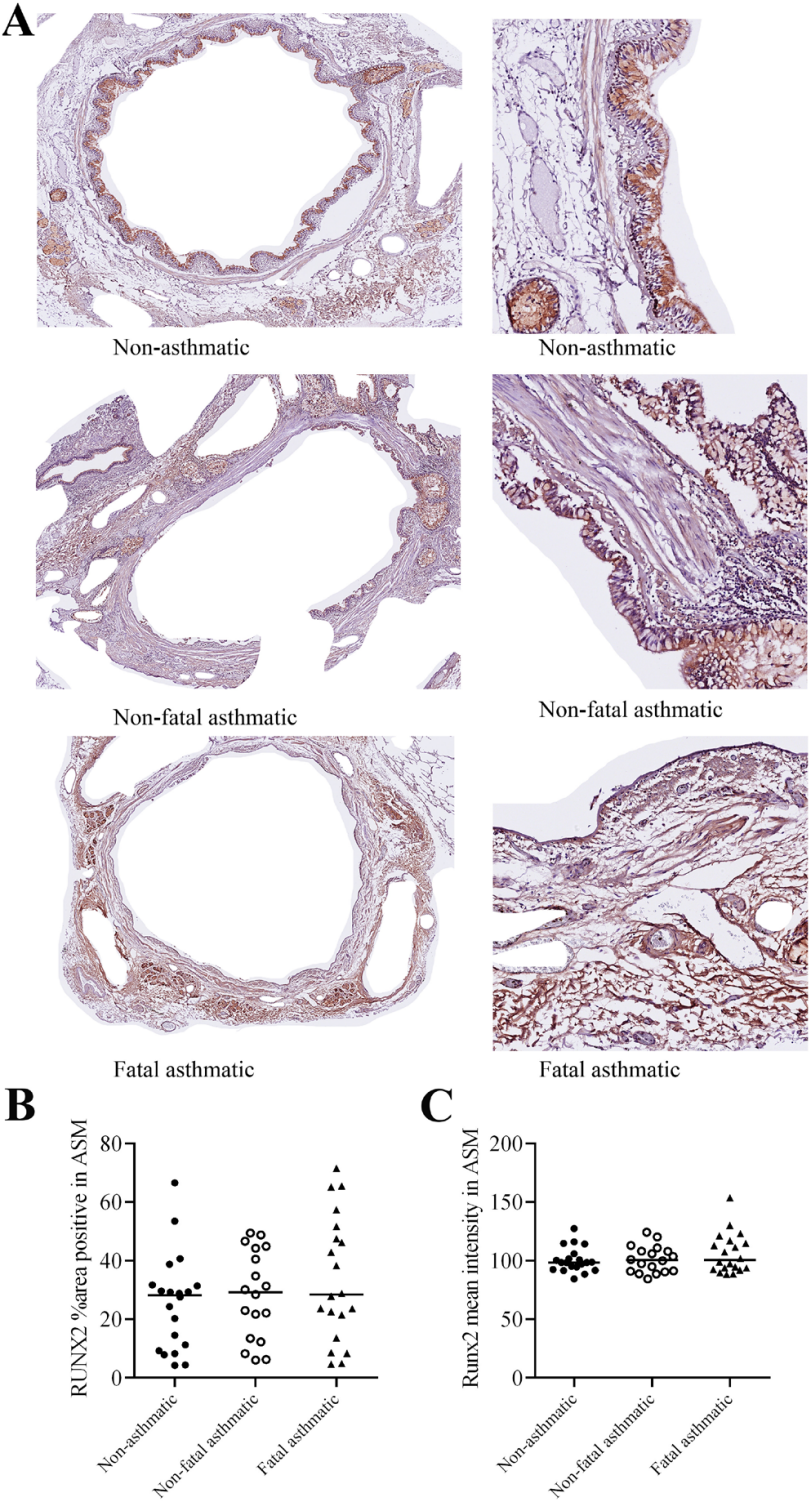
RUNX2 protein expression is variable in ASM in nonasthmatic and asthmatic airways. RUNX2 expression was detected in sections (4 μm) by immunohistochemistry (red‐brown color). Hematoxylin was used as a counterstain. Airway tissue from nonasthmatic (*n* = 10, 2 airways per donor), nonfatal asthmatic (*n* = 8, 2 airways per donor) and fatal asthmatic (*n* = 10, 2 airways per donor) patients were stained. The inset images are magnified to allow for assessment of RUNX2 expression in the airway smooth muscle within the wall. Representative images shown for each group (A, Scale bar = 100 μm). % ASM area positive for RUNX2 (B) and mean intensity of RUNX2 staining within the ASM (C) were quantified with Image J software. Each dot represents the ASM around 1 airway.

Immunohistochemistry for RUNX2 in lung tissues from nonasthmatic and asthmatic patients showed concentrated expression in the epithelial layer of airways independent of disease status (Figure 2A). Closer inspection of the airway wall (magnified boxes) revealed RUNX2 in the cytoplasm and nucleus of ASM cells in NA and A airways that were very variable in amount detected. There were no differences in the degree of RUNX2 staining detected in nonfatal versus fatal asthmatic airway tissues, with equally variable staining being observed in both tissue types.

### 3.3 TGF‐β1 Treatment Increases RUNX2 Only in NA‐ASM Cells

**FIGURE 3:**
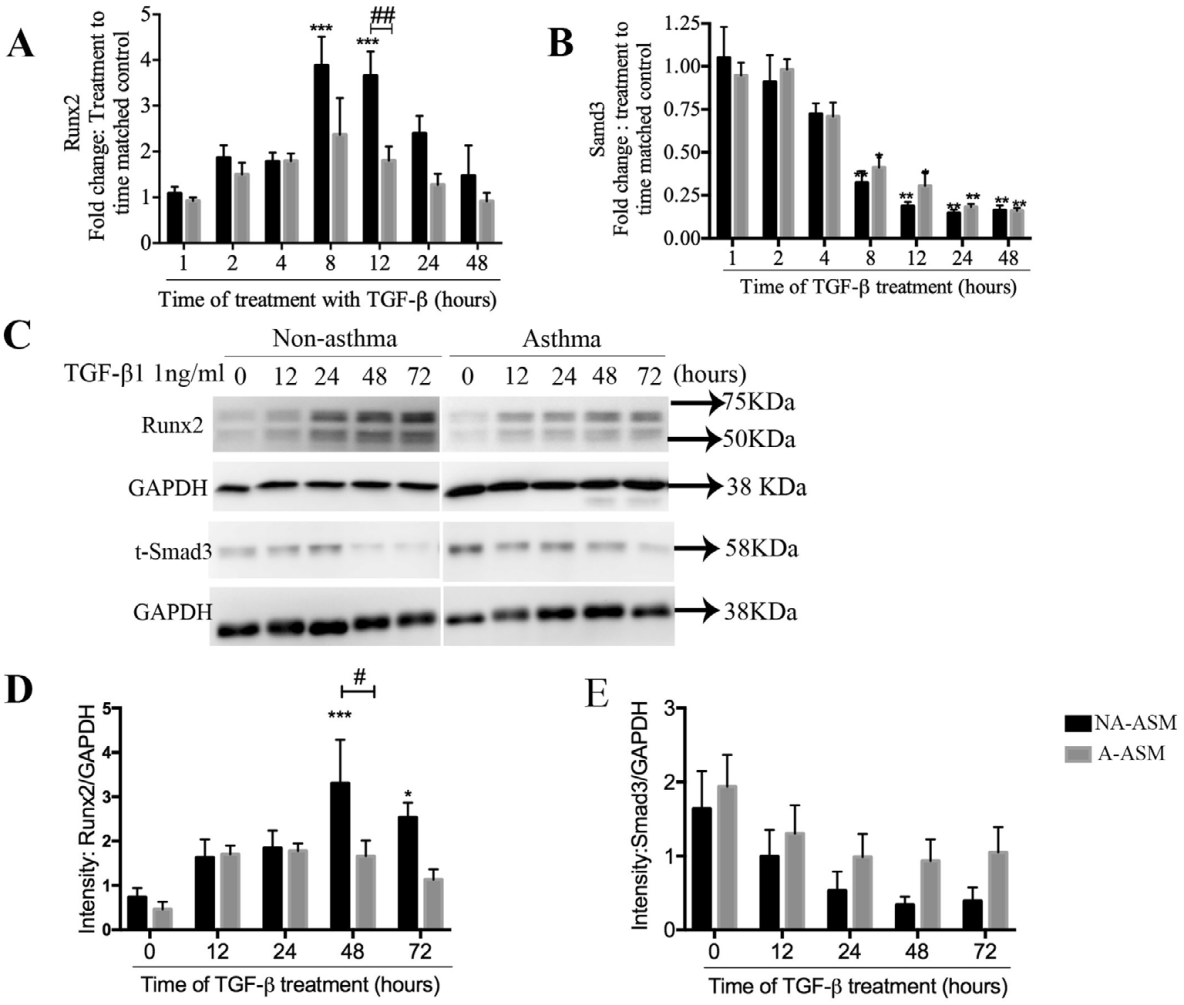
RUNX2 and SMAD3 are differentially regulated in A‐ and NA‐ASM cells. Levels of RUNX2 and SMAD3 were examined by PCR (A and B, respectively) and immunoblotting (C) in NA (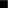) and A‐(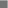) ASM cells. (D and E). Changes in protein expression were quantified using image J software. Data represent mean ± SD (*n* = 7 NA and *n* = 5 A). *p < 0.05, **p < 0.01, ***p < 0.001 denotes significance between BSA and TGF‐β. ^#^ *p* < 0.05, ^##^ *p* < 0.01, indicates significant difference between NA‐ and A‐ASM cells.

We next examined temporal regulation of *RUNX2* and *SMAD3* in NA‐ and A‐ASM cells. There was no difference in basal mRNA expression of *RUNX2* (Figure S1A, NA = 11, A = 12) or *SMAD3* (Figure S1B, NA = 4, A = 4). *RUNX2* mRNA levels in NA‐ASM (*n* = 7) cells increased 4‐fold after 8 (*p* < 0.001) and 12 (p < 0.001) hours of TGF‐β1 treatment (Figure 3A). In contrast, *RUNX2* mRNA levels were not modulated in A‐ASM by TGF‐β1 at any timepoint (Figure 3A). TGF‐β1 treatment longer than 8 h decreased *SMAD3* mRNA levels in both NA‐ (*n* = 4) and A‐ASM (*n* = 4) cells (Figure 3B). Changes in protein levels followed the mRNA expression (Figure 3C) with maximal RUNX2 protein detected in NA‐ASM cells after 48 h (*p* < 0.001; Figure 3D). Moreover, there was greater overall induction of RUNX2 protein in NA‐ASM (*n* = 7) than A‐ (*n* = 4) ASM cells (*p* < 0.01). The decline in SMAD3 protein level was less evident in A‐ (*n* = 5) compared to NA‐ASM (*n* = 7) cells (Figure 3E).

### 3.4 TGF‐β1 Selectively Increases RUNX2 Variant 1 in NA‐ASM

**FIGURE 4:**
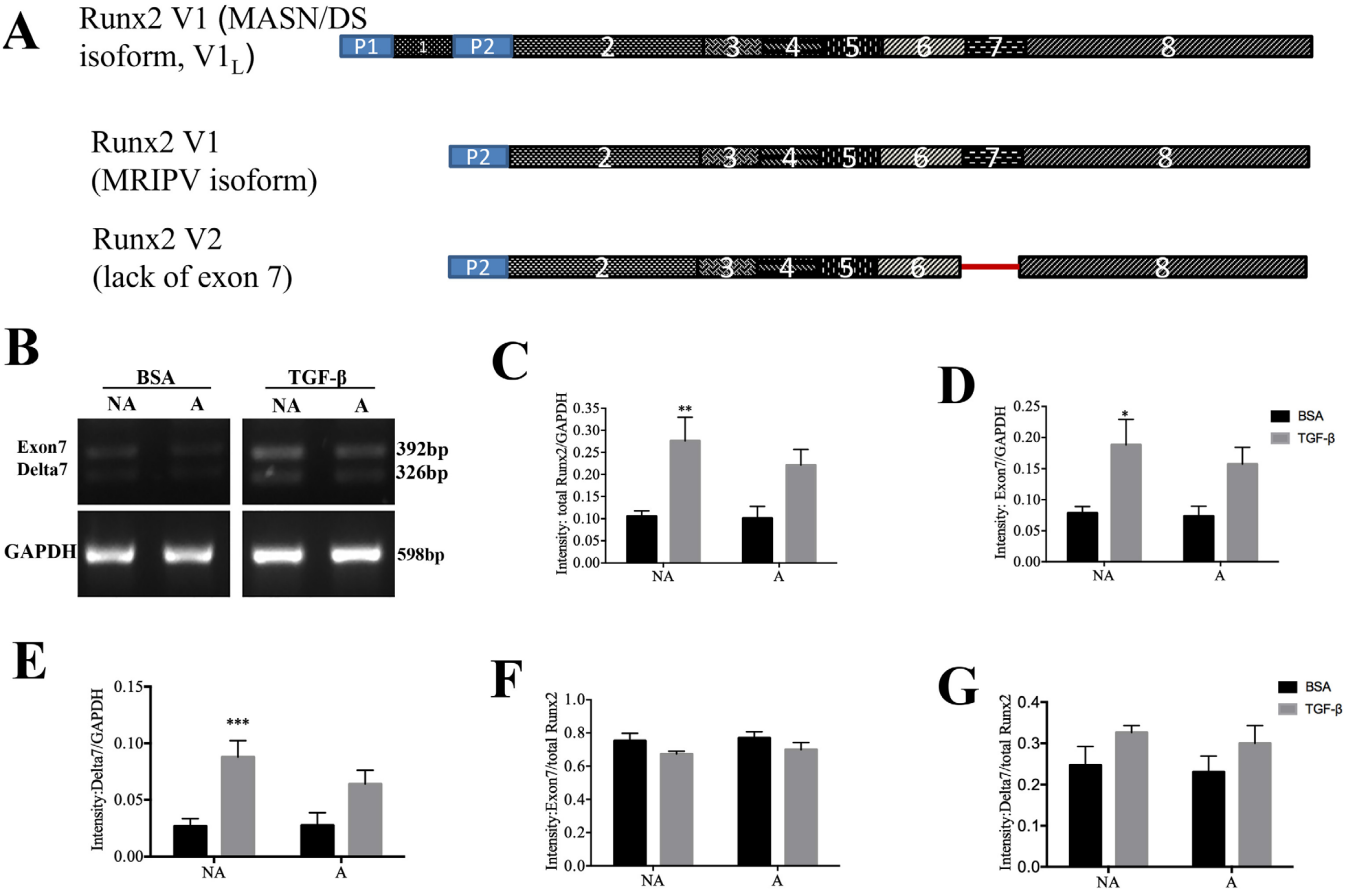
TGF‐β1 selectively increased RUNX2 variant 1 expression in NA‐ rather than A‐ ASM cells. (A) Schematic depicting exon usage resulting in different RUNX2 isoforms. Position of the primers used to detect Δ7 exon splice variants is shown. (B) Representative agarose gel showing PCR products for V1 (contains exon 7) and V2 (Δ7 exon) splice variants in NA‐ and A‐ASM cells with (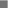) and without (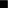) TGF‐β1 treatment. Densitometric analysis of (C) total *RUNX2*, (D) RUNX2 V1 and (E) RUNX2 V2 expression in NA‐ and A‐ ASM cells with TGF‐β1 treatment. Relative contribution of (F) V1 and (G) V2 to overall *RUNX2* expression in ASM cells. Data represent mean ± SD (*n* = 8 NA and *n* = 6 A). *p < 0.05, **p < 0.01, ***p < 0.001 denotes significance between BSA and TGF‐β1.

RUNX2 has multiple isoforms derived from differential splicing of its 8 exons and differential promoter utilization (Figure 4A). The immunohistochemical staining we performed on the lung tissue sections was not able to differentiate between these isoforms. Splice variants (V) containing exon 7 (V1 and V1 long) attenuate the biological effects of TGF‐β1 via translocation to the nucleus and antagonizing SMAD signaling. To investigate differences in *RUNX2* isoform expression between NA‐ and A‐ASM cells we conducted PCR with primers spanning exon 7 (Figure 4A). *RUNX2* isoforms with (V1) and without (V2) exon 7 were expressed in similar ratios basally in both NA‐ and A‐ASM cells; however, V1 isoforms occurred in greater abundance (Figure 4B). Similar to our qPCR data, TGF‐β1 selectively increased total *RUNX2* expression in NA‐ASM cells (Figure 4B,C, NA = 8 and A = 6, *p* < 0.01). Variant 1 (Figure 4D) and V2 (Figure 4E) abundance increased in NA‐ASM (*n* = 8, *p* < 0.05) after TGF‐β1 stimulation; however, the proportion of V1 and V2 transcripts in the total RUNX2 pool remained constant in both NA‐ and A‐ASM (Figure 4F,G). Thus, the proportion of “active” RUNX2 did not change but the overall amounts increased in NA‐ASM.

In addition to Exon 7, differential promoter utilization in the *RUNX2* gene (promoter 1 (P1) and 2 (P2)) (Figure S2A) results in the inclusion of an additional exon at the N‐terminus (exon 1.1, Figure 4A), which may change protein properties. PCR analysis indicated that both P1 and P2 were used to generate transcripts in ASM cells (Figure S2B,C); however, no differences in P1 and P2 utilization were observed between NA‐ and A‐ASM basally or after TGF‐β1 stimulation (Figure S2B–E), suggesting that biased promoter utilization did not account for the reduced induction of *RUNX2* in A‐ASM cells.

### 3.5 Nuclear Translocation of SMAD3 Is Greater in A‐ASM Compared to NA‐ASM Cells After TGF‐β1 Treatment

**FIGURE 5:**
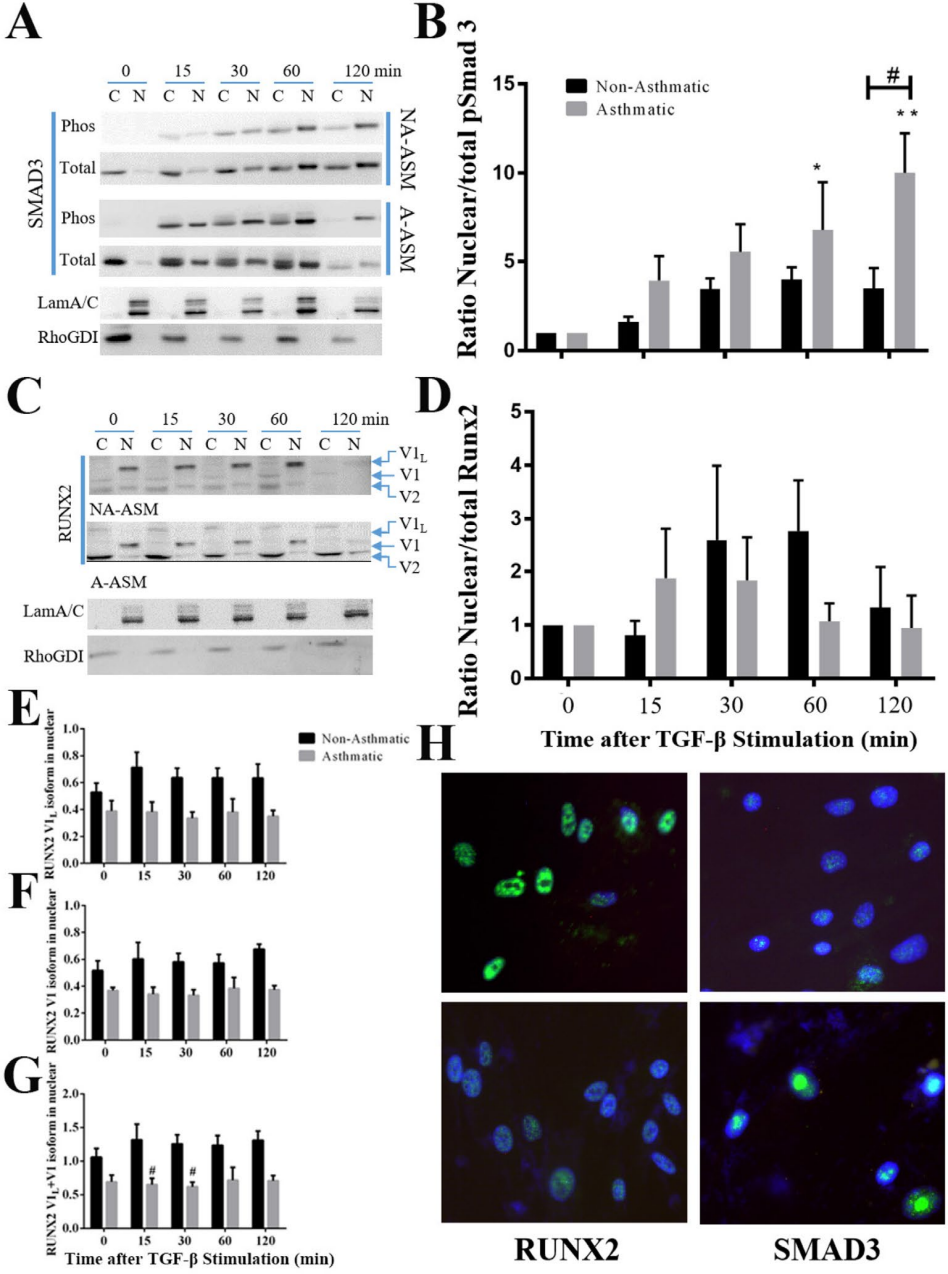
TGF‐β1‐induced nuclear translocation of SMAD3 is greater in A‐ASM. Subcellular fractionation of ASM cells was used to examine changes to nuclear (N) and cytoplasmic (C) pools of (A) SMAD3 and (C) RUNX2 in response to TGF‐β1 treatment (1 ng/mL) in NA‐ (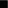) and A‐(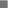) ASM cells. Blots were quantified for (B) nuclear/total pSmad3, (D) nuclear/total RUNX2, (E) V1_L_ in nuclear fraction, (F) V1 in nuclear fraction and (G) V1_L_ + V1 in nuclear fraction. Data represent mean ± SD (*n* = 7 NA and *n* = 5 A). *p < 0.05, **p < 0.01, denotes significance between BSA and TGF‐β1. ^#^ *p* < 0.05 indicates significant difference between NA‐ and A‐ASM cells. (H) Representative images from immunofluorescent analysis of subcellular localization of RUNX2 and SMAD3 in NA‐ and A‐ASM cells (*n* = 3 of each).

In response to TGF‐β1 signaling SMAD3 is phosphorylated and translocated to the nucleus; however, a previous study reported that a RUNX2‐SMAD3 complex repressed TGF‐β1 signaling [25]. By fractionating ASM we showed that TGF‐β1 stimulation induced SMAD3 phosphorylation which preceded translocation from cytoplasm to nucleus in ASM cells (Figure 5A,B), with greater pSMAD3 nuclear translocation in A‐ASM compared to NA‐ASM (*p* < 0.05). Three RUNX2 isoforms were detectable in ASM cell lysates basally (Figure 5C). Of these, the V2 variant (50 kDa) was primarily cytoplasmic in both NA‐ and A‐ASM cells. In NA‐ASM V1 (60kDa) localized in the cytoplasm while V1_L_ (75 kDa) variants were exclusively nuclear. Strikingly, the localization of V1 and V1_L_ were reversed in A‐ASM (Figure 5C). We did not observe differences in the translocation of RUNX2 variants into the nucleus following TGF‐β1 treatment (Figure 5D) in either NA‐ (*n* = 7) or A‐ASM (*n* = 5) cells. However, after quantifying V1_L_ and V1 isoform expression in the nuclear fraction, we observed that while there was no difference in the expression of V1_L_ (Figure 5E) or V1 (Figure 5F) alone between A‐ and NA‐ASM, the sum combination of V1_L_ + V1 expression in the nuclear fraction was higher in NA‐ than A‐ASM at 15 and 30 min after TGF‐β1 treatment (Figure 5G). These findings were supported by immunofluorescent photomicrographs of the cells (Figure 5H). The differential subcellular localization of the RUNX2 isoforms, and the differential localization between NA‐ and A‐ASM cells, may contribute to the dysregulation of TGF‐β1 signaling.

### 3.6 Overexpression of RUNX2 Variants Modulates Markers of ECM and ASM Remodeling but Not of Inflammation or Angiogenesis in A‐ASM Cells

**FIGURE 6:**
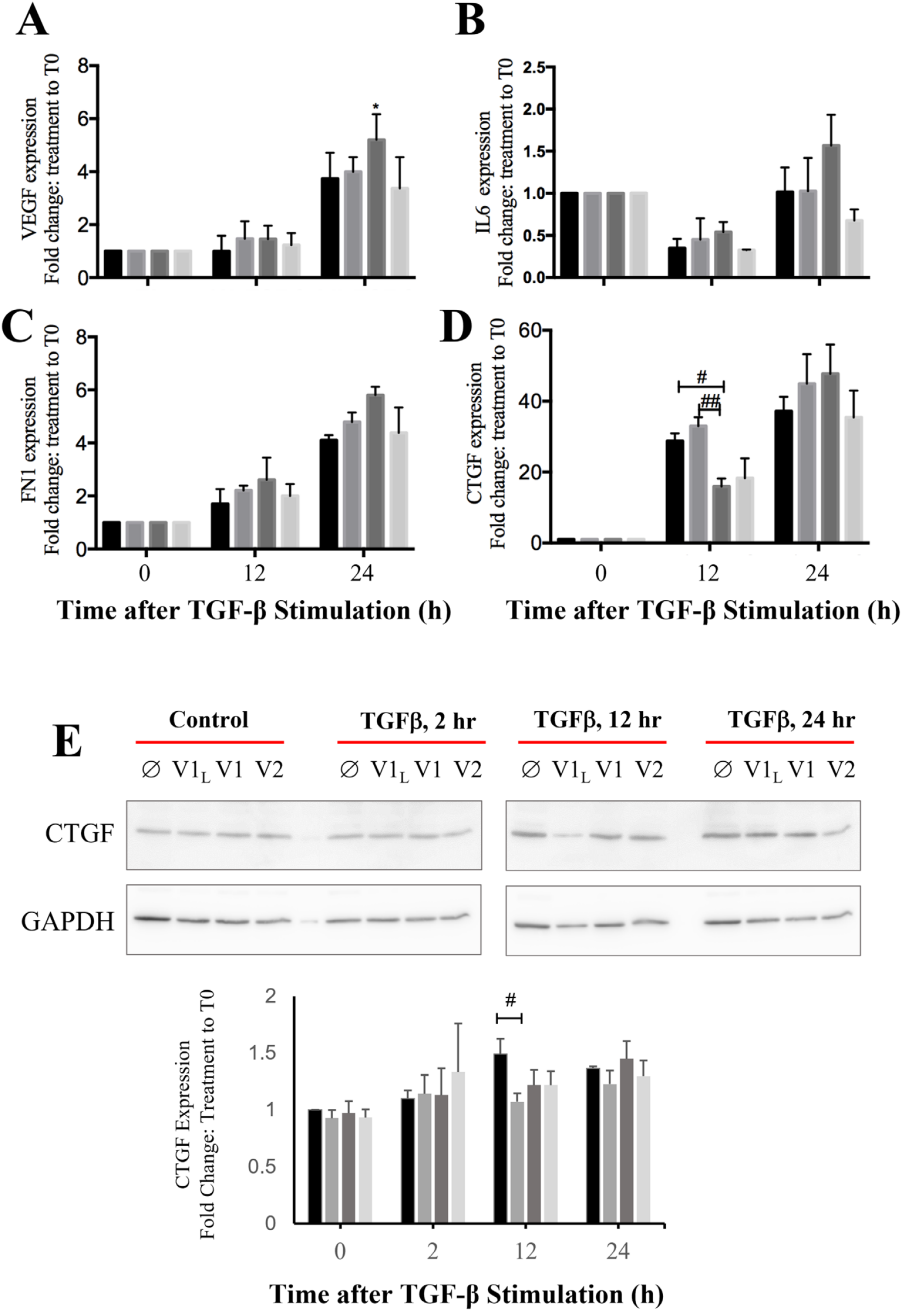
Restoration of RUNX2 variant 1 expression reverses ECM remodeling in A‐ASM cells. The effect of RUNX2 isoforms on markers of angiogenic potential (A, *VEGF‐A*), and inflammation (B, *IL‐6*) or ECM remodeling (C, Fibronectin, *FN1*; D, *CTGF*) was assessed in iA‐ASM cells. Changes in CTGF expression due to RUNX2 variant overexpression in iA‐ASM were also assessed by immunoblotting (E; representative images). Data represent mean ± SD (*n* = 3). *p < 0.05 denotes significance between BSA and TGF‐β. ^#^ *p* < 0.05, ^##^ *p* < 0.01 indicates significant difference between empty vector (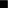) and the RUNX2 variants V1_L_ (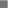), V1 (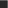), and V2 (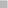) in iA‐ASM cells.

To study the effects of different RUNX2 variants on asthma, we transfected iA‐ASM cells with expression vectors containing the coding sequence for *RUNX2‐V1*, ‐*V1*_*l*_ and ‐*V2*. qPCR and immunoblotting (Figure S3A,B, respectively) confirmed the overexpression of RUNX2 isoforms, and the impact on biological and molecular responses that are considered to be pro‐remodeling/inflammation was assessed. No differences in *CTGF*, *FN‐1*, *VEGF‐A*_*165*_, or *IL‐6* mRNA abundance were detected following overexpression of *RUNX2* variants alone (Figure S4A–D). Similarly, the induction of *FN1*, *VEGF‐A*_*165*_, or *IL‐6* mRNA by TGF‐β1 was not diminished by any RUNX2 variant (Figure 6A–C). Notably, the induction of *CTGF* mRNA by TGF‐β1 was blunted by *RUNX2‐V1* and *RUNX2‐V2* expression at 12 h (*p* < 0.05; Figure 6D) which was consistent with other studies [25]. The RUNX2‐V1_l_ variant had no influence on *CTGF* mRNA levels. Conversely, all three RUNX2 isoforms suppressed CTGF protein at 12 h of TGF‐β1 stimulation suggesting regulation through transcriptional control (V1 and V2) and protein stability (V1_l_) may be involved (Figure 6E).

**FIGURE 7:**
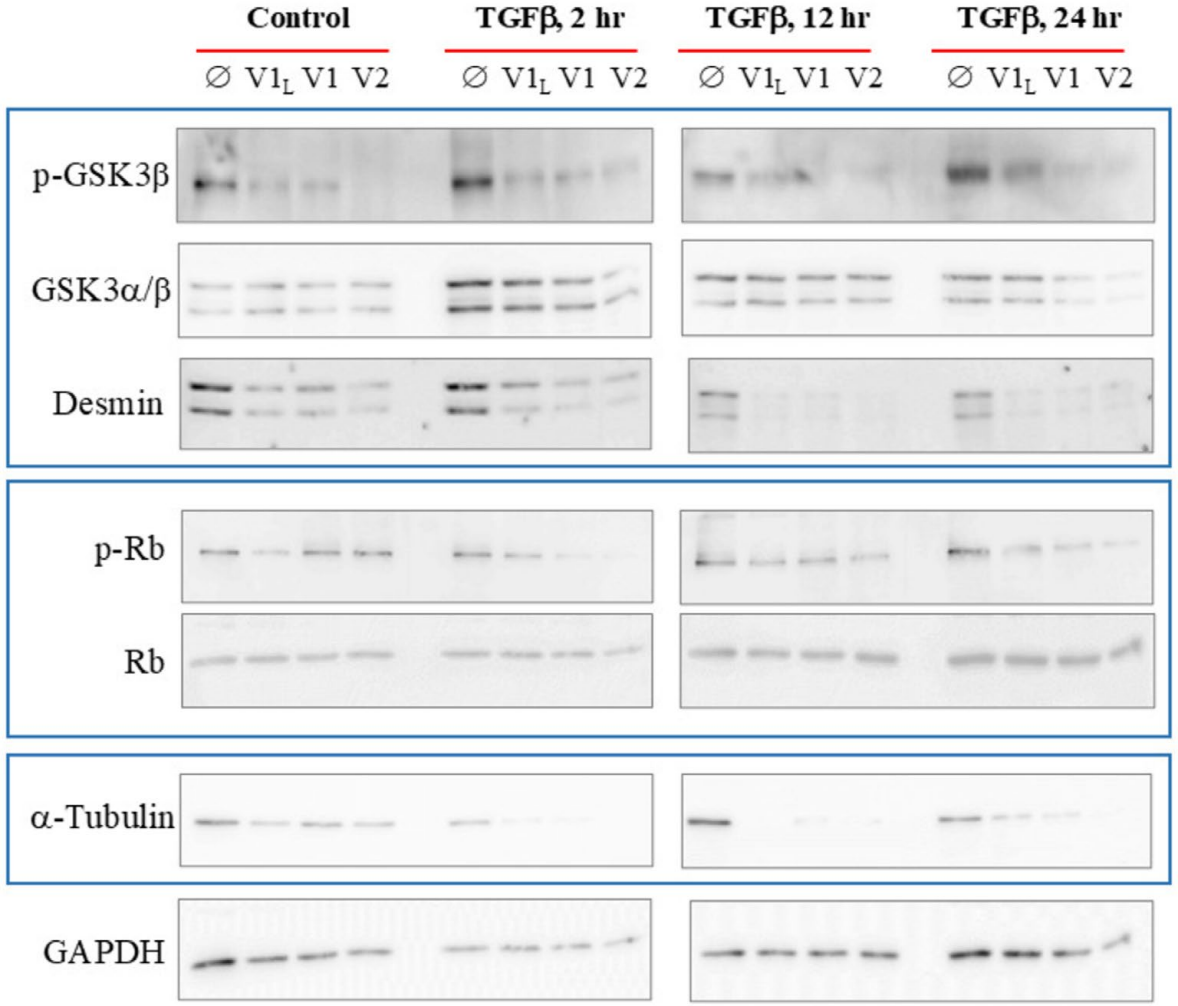
RUNX2 isoforms blunt hypertrophic and hyperplastic responses in A‐ASM cells. iA‐ASM cells were transfected with empty vector (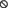), RUNX2 V1_L_, RUNX2 V1 or RUNX2 V2 and stimulated with TGF‐β1 (1 ng/mL) for up to 24 h. Expression of markers of proliferation (p‐Ser^795^/total pRB), hypertrophy (p‐Ser9/total GSK 3β, desmin) and cytoskeletal integrity (α‐tubulin) were determined by immunoblot. Representative images, for *n* = 3 replicates, of immunoblots are shown.

We next investigated the contribution of RUNX2 isoforms to ASM remodeling. Overexpression of all RUNX2 isoforms at baseline abrogated phosphorylation of GSK‐3β at Ser^9^, indicating increased GSK‐3β activation, and diminished desmin expression (Figure 7). These changes in key regulators of ASM hypertrophy [43, 44] continued across all timepoints with TGF‐β1 stimulation. Restoration of RUNX2 isoform expression in A‐ASM cells also prevented TGF‐β1‐induced cell cycle progression as evident by decreased pSer^795^ Rb levels (Figure 7). Moreover, the overexpression of all RUNX2 isoforms in A‐ASM cells downregulated α‐tubulin, an important protein for cell division and hypertrophic growth [45, 46]. Collectively, these data suggest that the loss of RUNX2 in A‐ASM cells is a seminal event contributing to the increased ASM cell bulk in the airways.

### 3.7 ASM From Asthmatic Patients Displays a Greater Degree of RUNX2 Splicing in Bronchial Biopsies

**FIGURE 8:**
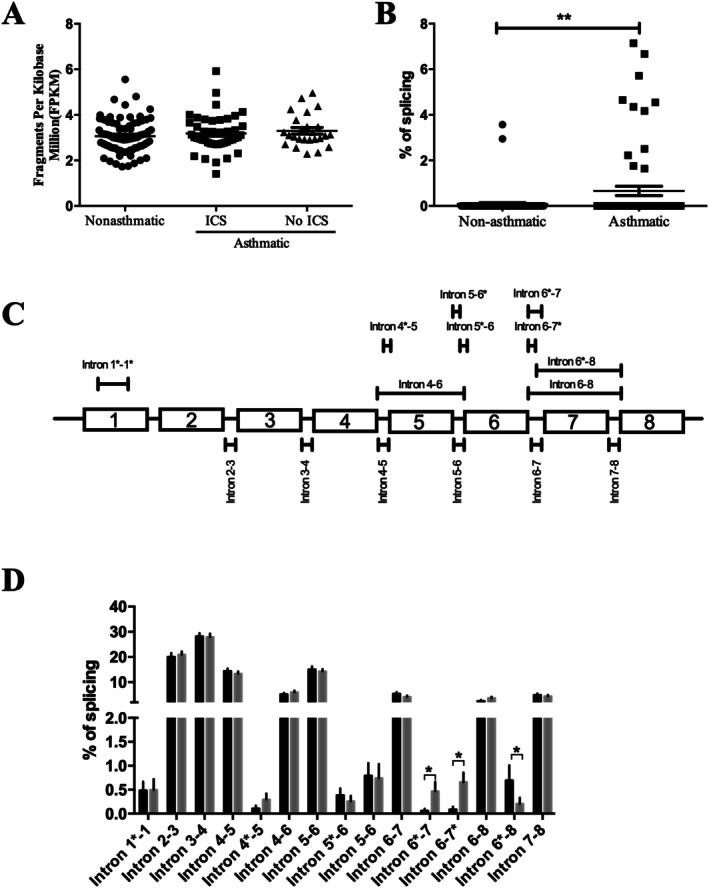
ASM from asthmatic patients display a greater degree of RUNX2 splicing in human bronchial biopsies. (A) *RUNX2* mRNA expression (in fragments per kilobase million; FPKM) and (B) percentage alternative splicing was assessed in bronchial biopsies from healthy controls (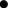, *n* = 77) and asthmatic patients using inhaled corticosteroids (ICS) (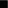, *n* = 44) or not (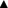, *n* = 25). (C) Schematic of the alternative splicing events discovered in the RNAseq analysis of asthmatic airway biopsies. (D) Relative prevalence of each of the splicing events found in airway biopsies from healthy controls (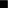) and asthmatic patients (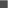). Data represent mean ± SD. *p < 0.05, ** *p* < 0.005 indicates significant difference between healthy controls and asthmatic patients.

To determine whether any RUNX2 isoforms might contribute to the disease process in patients we assessed whether *RUNX2* expression was altered in asthmatic bronchial biopsies compared to healthy subjects. No difference was found for total *RUNX2* abundance between the two groups (*p* > 0.05, Figure 8A); however, *RUNX2* is plenteous in epithelial cells (Figure 2) and biopsies contain a mix of cell types including epithelial and ASM cells, potentially masking cell type specific differences. When examining *RUNX2* transcripts, alternatively spliced forms were far more common in biopsies from asthmatic patients than healthy subjects (Figure 8B). The locations of the splicing events detected in our study (Figure 8C) were more comprehensive and complex than the limited public databases. Indeed, the splicing variants identified here (Figure 8C,D) comprise a significant proportion of transcripts with the ΔExon 5 (intron 4–6) and ΔExon 7 (intron 6–8) variants occurring frequently. Despite their higher frequency the ΔExon 5 and ΔExon 7 variants were not significantly different between healthy and asthmatic patients; however, the intron 6*‐7 and intron 6‐7* splicing events were more frequent in asthmatic airways while the intron 6*‐8 splice variant was more frequent in healthy airways.

## 4 Discussion

This is the first study to illuminate the regulation and function of RUNX2 with respect to asthma pathobiology. While detection of RUNX2 protein was highly variable in airway tissues, we revealed lower *RUNX2* gene expression in asthmatic airway cells and elucidated this deficiency amplifies TGF‐β1‐induced SMAD3 responses in A‐ASM cells. RUNX2 variants attenuated TGF‐β1‐induced CTGF expression and markers of hypertrophic/proliferative responses in A‐ASM suggesting a role in airway homeostasis. There was also a higher rate of alternative exon usage in *RUNX2* mRNA in bronchial biopsies of asthmatic patients, suggesting that the levels are not only changed in asthma but also that the coding sequence is potentially altered, yielding truncated or unstable proteins.

The loss of specific RUNX2 isoforms may potentially be a fundamental event in the loss of airway homeostasis that promotes remodeling in asthma. TGF‐β1 stimulation increased *RUNX2* transcript levels only in NA‐ASM cells. Whilst the diversity of undocumented alternatively spliced events in RUNX2 in asthmatic biopsies leaves questions over the roles of these new altered forms in disease one consequence may be protein instability. This could explain the decrease in RUNX2 protein when expression of the primary E3 ligase (WWP1) is simultaneously reduced in A‐ASM. Although individual events only occurred with relative infrequency (< 2% of total reads), their impact on pathogenesis may be significant especially given RUNX2 haplodeficiency is sufficient to cause cleidocranial dysplasia [47]. Moreover, splicing changes affecting inclusion of Exon 7 (6*‐7 and 6‐7*) would impact RUNX2 function through altered heterotypic interactions with cognate binding partners (such as HDAC6, SMAD, YAP). However, splicing events did not seem to vary greatly between asthma‐ICS and asthma‐no–ICS (Figure 8A) suggesting they are not influenced by therapeutic exposures.

To explore a pathogenic role for the loss of RUNX2 in asthma we sought to restore normal responses to A‐ASM by overexpression of RUNX2 variants in immortalized cells. Of the variants explored, only V1 repressed TGF‐β1‐induced *CTGF* mRNA levels, which is consistent with other studies in human aortic vascular smooth muscle cells (HASMCs) [25]. This finding indicates that RUNX2‐V1 may be important in asthma since CTGF is associated with basement membrane thickness in asthmatic patients [15, 42, 48]. Activation of GSK‐3β and desmin are important regulators of cell growth, both of which are elevated in models of allergic airway disease and correlate with poor clinical outcomes [43, 44]. The suppression of these pathways by all RUNX2 variants (unlike CTGF) is further evidence of the role of RUNX2 in maintaining airway homeostasis.

The three dominant isoforms of *RUNX2* (V1, V1_L_, and V2) appear to differ between NA‐ and A‐ASM cells. All studies to date have stressed the requirement of exon 7 for RUNX2 functionality; puzzlingly, nuclear localization of isoforms containing exon 7 (V1 and V1_L_) differed between NA‐ and A‐ASM cells. V1_L_ and V1, which were considered to have an “active” role in RUNX2 functional effects, were higher in NA‐ASM nuclear fractions compared to A‐ASM after TGF‐β1 stimulation. These data suggest some level of functional redundancy between the Exon 7 containing variants. The role of the N‐terminal extension in V1_L_ in regulating subcellular localization has not been well explored. However, it is unlikely to be due to the induction of inhibitors, such as MSX2, as these were unchanged between NA‐ and A‐ASM. Conversely, V2 was largely cytoplasmic in both cell types, confirming the importance of Exon 7 in promoting nuclear‐cytoplasmic shuttling [23, 24].

The DNA binding capacity of RUNX2 isoforms did not seem to influence the regulation of ASM hypertrophy/hyperplasia. Classical TGF‐β1 signaling involves phosphorylation and nuclear translocation of SMADs, where they bind the SMAD binding element (SBE) in the target gene and mediate transcription [49]. The kinetics of RUNX2 translocation did not correlate with SMAD3 after TGF‐β1 treatment, making a direct interaction of the two improbable. A study in HASMCs also showed an indirect mechanism for the inhibition of SMAD3 by a RUNX2‐P300/CBP (cyclic AMP receptor element binding protein [CREB] binding protein) or HDAC complex [25]. A recent study had shown that TGF‐β1 promoted RUNX2 translocation through protein kinase A (PKA) [50] with activation by TGF‐β1 being SMAD‐dependent [51]. Consequently, the mechanism for reversing the ASM hypertrophy/hyperplasia is likely through interaction with co‐factors, such as HDACs, which in our system were unchanged between NA‐ and A‐ASM, and did not appear to be influenced by the presence of Exon 7.

Examination of human lung tissue revealed that RUNX2 was abundant in the epithelial cell layer and to a lesser extent in ASM cells, which is consistent with a recent study in idiopathic pulmonary fibrosis (IPF) [52]. In that study, RUNX2 was expressed in several cell types in IPF lungs, including strong signals in fibrotic alveolar type II (ATII) cells, with less expression in myofibroblasts. Knocking down RUNX2 decreased profibrotic ATII proliferation and migration but increased ECM expression in fibroblasts, suggesting that the role of RUNX2 is opposite between the two cell types. Another recent study showed the *RUNX2* transcript was increased in bronchial brushing from asthmatic compared with control patients and epithelial *RUNX2* expression positively correlated with eosinophils in induced sputum [53]. However, normal airways and NA‐ASM cells maintain higher RUNX2 levels, suggesting RUNX2 suppresses cell growth and hypertrophy in ASM of healthy airways, the absence of which may reflect a contributory change driving asthma pathogenesis. This is reflected in the vasculature where enhanced RUNX2 in vascular SMC negatively regulates CTGF expression, suppresses growth and induces apoptosis in endothelial cells [25]. The tools available for studying RUNX2 protein expression do not allow for the identification of the RUNX2 variants in tissues, where the RUNX2 protein detected in the asthmatic airways in this study may be the V2 form which lacks the functional Exon 7.

In conclusion, we showed for the first time that TGF‐β1 selectively increased RUNX2 only in NA‐ASM cells but not A‐ASM cells. Suppression of CTGF expression via RUNX2 may afford novel therapeutic opportunities for asthma in the future.

## Author Contributions

Janette K. Burgess and Anthony W. Ashton designed the research, supervised the project, reviewed and edited the manuscript. Junfei Wang conducted experiments, performed data analysis for cell experiments, and wrote the manuscript. Alen Faiz performed data analysis for RUNX2 splicing in bronchial biopsies. Theo Borghuis performed the immunohistochemical staining and digital image analyses. Rob van de Velde conducted immunofluorescence experiments. All authors were involved in revising the manuscript and approved the final version for submission.

## Funding

This work was supported by the National Health and Medical Research Council (NHMRC) (#1061712 and #1032695), European Union and University of Groningen, Nederlandse Organisatie voor Wetenschappelijk Onderzoek (NWO) (Aspasia 015.013.010), and MOST | National Natural Science Foundation of China (NSFC) (82000025).

## Conflicts of Interest

The authors declare no conflicts of interest.

## Supporting information


**Figure S1:** fsb271544‐sup‐0001‐FigureS1.pdf.

**Figure S2:** fsb271544‐sup‐0002‐FigureS2.docx.

**Figure S3:** fsb271544‐sup‐0003‐FigureS3.pdf.

**Figure S4:** fsb271544‐sup‐0004‐FigureS4.pdf.

**Figure S5:** fsb271544‐sup‐0005‐FigureS5.pdf.

**Table S1:** fsb271544‐sup‐0006‐TableS1.pdf.

**Table S2:** fsb271544‐sup‐0007‐TableS2.pdf.

## Data Availability

All relevant data are within the manuscript and its supplemental files.
