# Supplementary material for: Runx2 Regulated Airway Homeostasis Is Disrupted in Asthma
Source: FASEB J. 2026 Feb 17;40(4):e71544. doi: 10.1096/fj.202502088R (PMC12911552; doi:10.1096/fj.202502088R)
Supplement: Supplementary file 1 — Figure S1: fsb271544‐sup‐0001‐FigureS1.pdf. [file FSB2-40-e71544-s005.pdf]

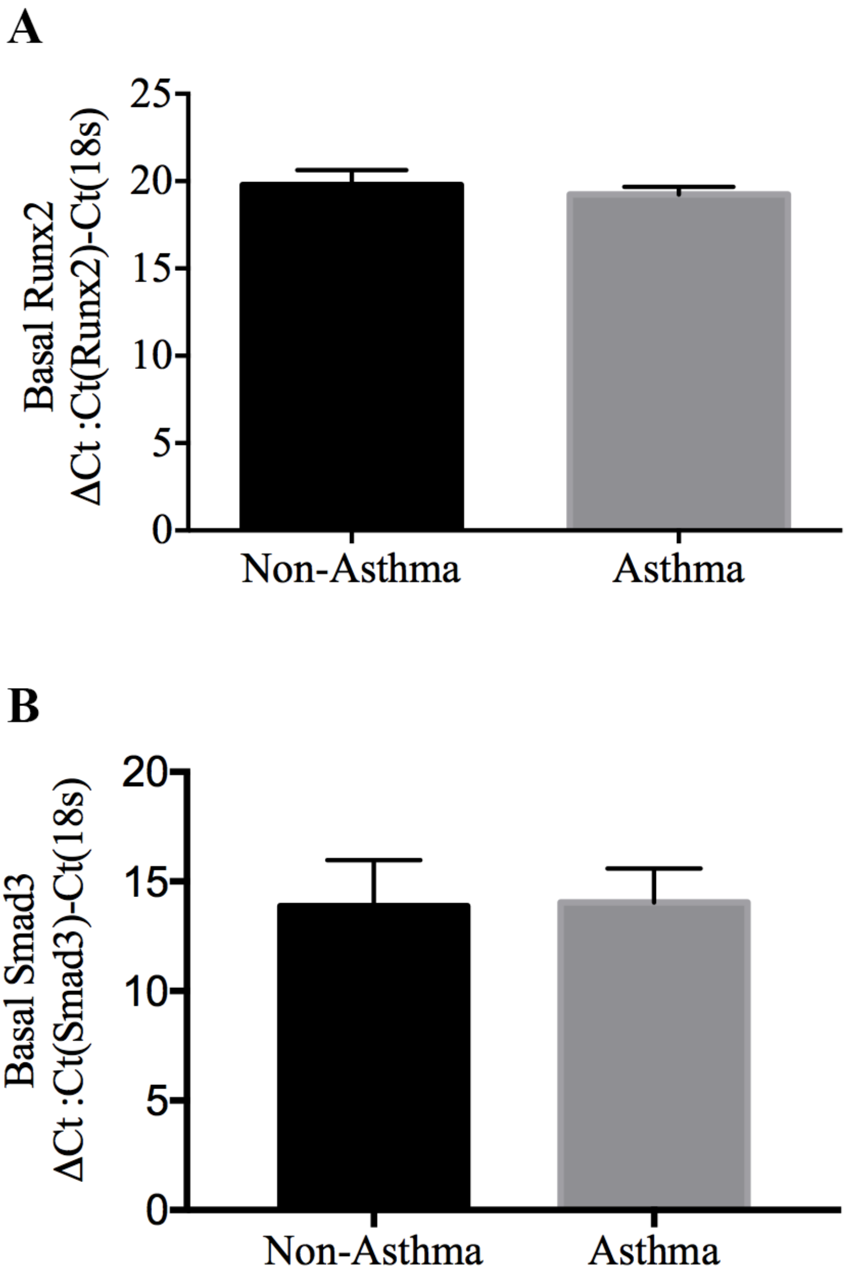

Supplementary Figure 1 Basal expression of Runx2 and Smad3 is similar in NA- and A-ASM cells. Quantitative RT-PCR was used to assess expression of **a)** Runx2 and **b)** Smad3 in unstimulated NA-(■) and A-(■) ASM cells. Data represent mean±SD (n=7 NA and n=5 A).
