# Supplementary material for: Runx2 Regulated Airway Homeostasis Is Disrupted in Asthma
Source: FASEB J. 2026 Feb 17;40(4):e71544. doi: 10.1096/fj.202502088R (PMC12911552; doi:10.1096/fj.202502088R)
Supplement: Supplementary file 2 — Figure S2: fsb271544‐sup‐0002‐FigureS2.docx. [file FSB2-40-e71544-s003.docx]

**
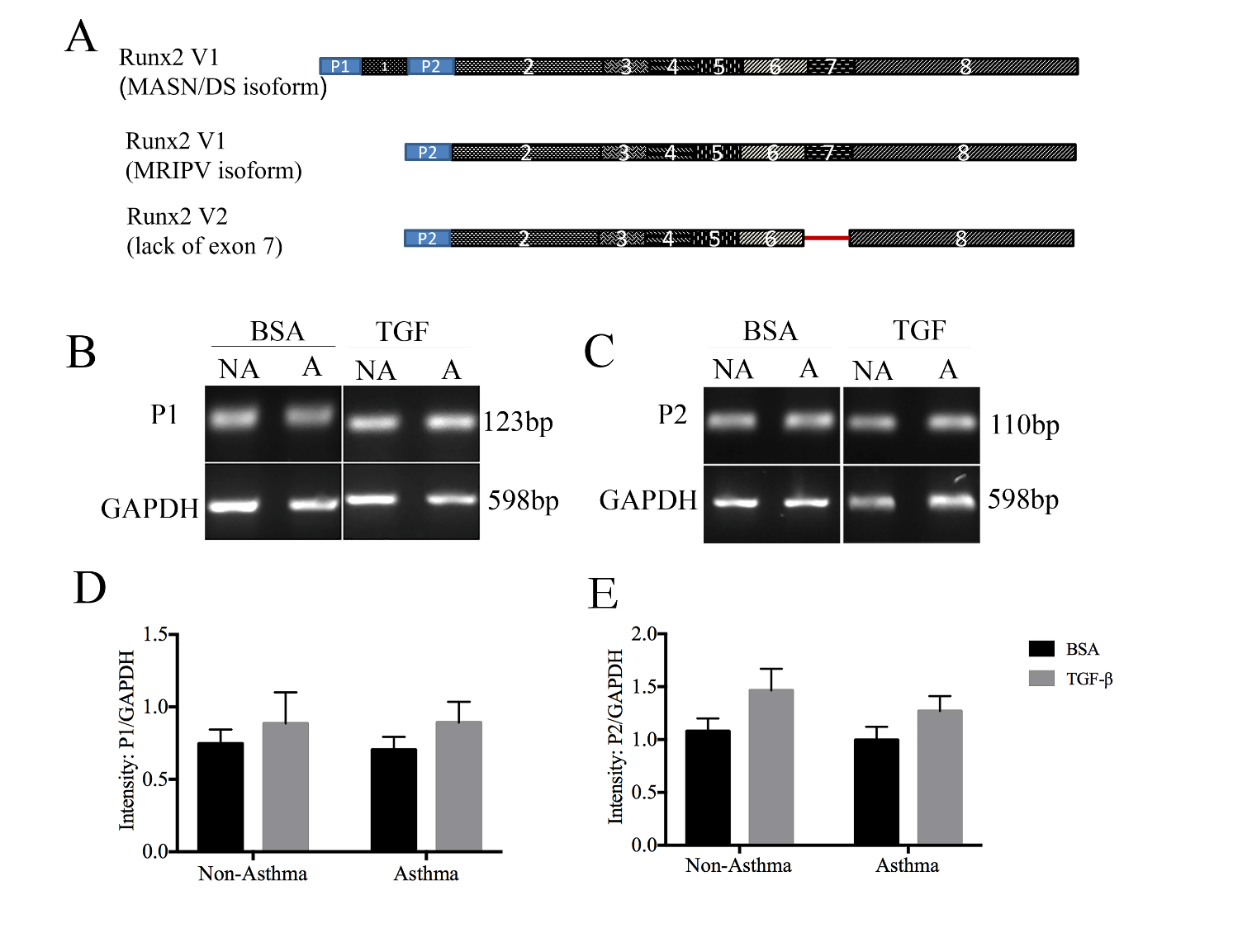
**

**Supplementary Figure 2** Promoter utilization for Runx2 does not differ between NA- and A-ASM cells. **a**) Schematic depicting the exon usage resulting in the different Runx2 isoforms. Additional exon 1.1 on the long form results from differential promoter usage. Representative agarose gel showing the PCR products for **b)** P1 and **c)** P2 usage in NA- and A-ASM cells with and without TGF-β treatment. Densitometric analysis of **d)** P1 and **e)** P2 expression in NA- (■) and A- (■) ASM cells with and without TGF-β treatment. Data represent mean±SD (n=7 NA and n=5 A). Gel images are representative of n=5 independent experiments.
