# Supplementary material for: Runx2 Regulated Airway Homeostasis Is Disrupted in Asthma
Source: FASEB J. 2026 Feb 17;40(4):e71544. doi: 10.1096/fj.202502088R (PMC12911552; doi:10.1096/fj.202502088R)
Supplement: Supplementary file 3 — Figure S3: fsb271544‐sup‐0003‐FigureS3.pdf. [file FSB2-40-e71544-s001.pdf]

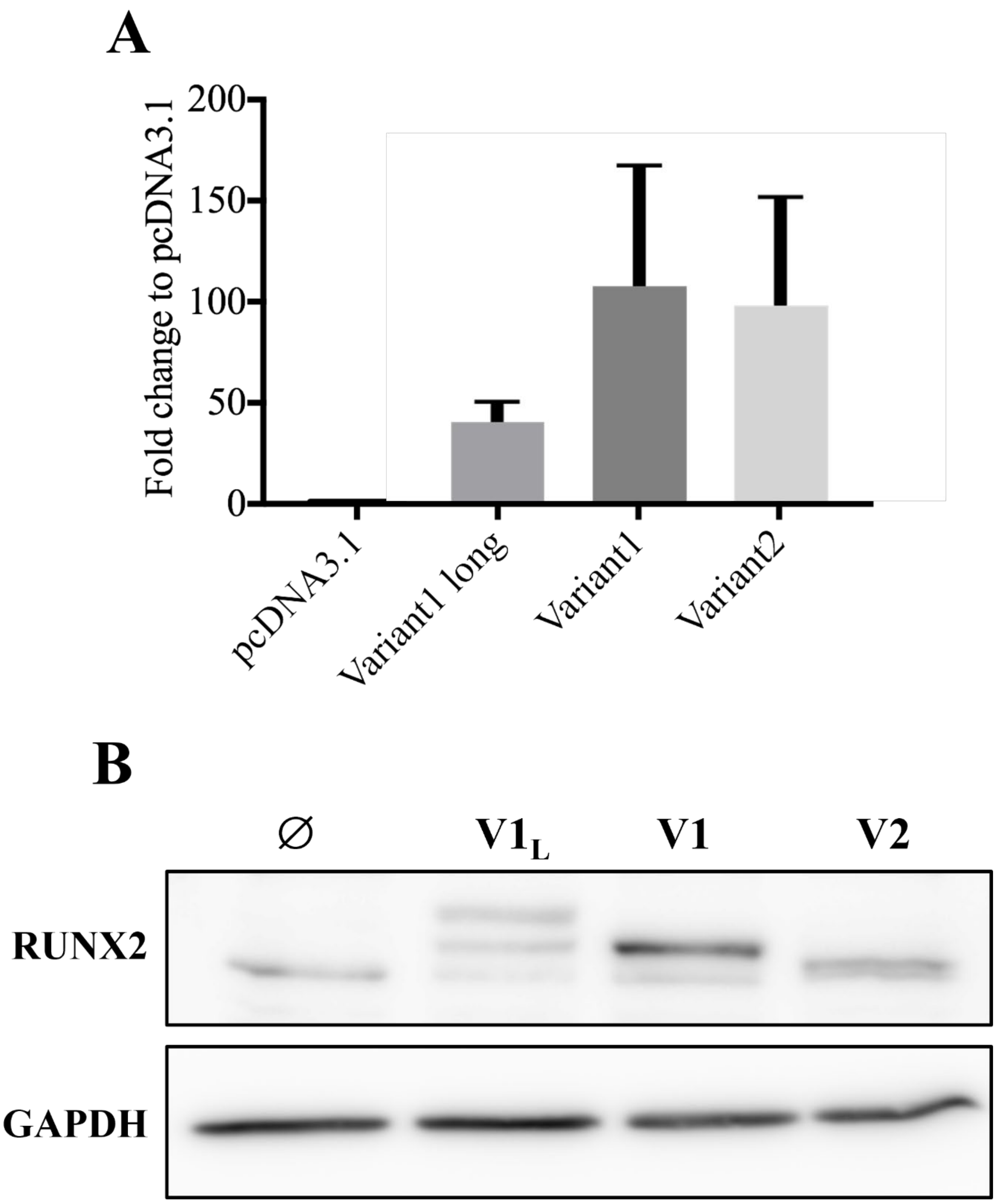

Supplementary Figure 3 Runx2 overexpression in i**A-ASM cells.** Immortalized A-ASM cells were transfected with plasmids encoding V1 (■), V1L (■) and V2 (■) and expression documented using qRT-PCR (**A**) and immunoblotting (**B**). Empty vector (Ø, pcDNA3.1) was used as a control. Data represent mean±SD (n=3). Images are representative of n=3 independent samples.
