# Supplementary material for: Runx2 Regulated Airway Homeostasis Is Disrupted in Asthma
Source: FASEB J. 2026 Feb 17;40(4):e71544. doi: 10.1096/fj.202502088R (PMC12911552; doi:10.1096/fj.202502088R)
Supplement: Supplementary file 4 — Figure S4: fsb271544‐sup‐0004‐FigureS4.pdf. [file FSB2-40-e71544-s007.pdf]

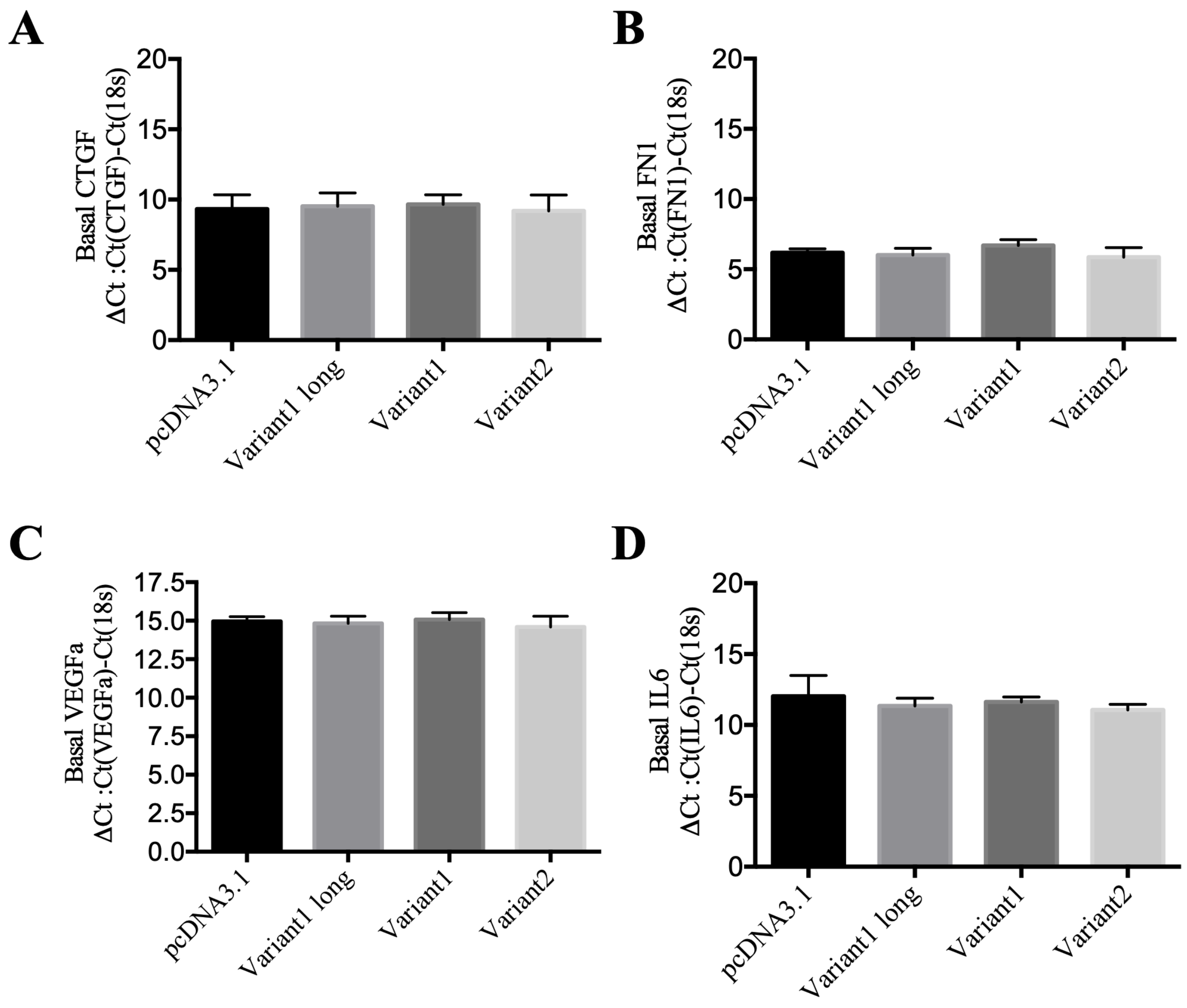

Supplementary Figure 4: Runx2 isoforms do not affect basal expression in ASM cells. The effect of Runx2 isoforms on ECM remodeling (**a**, CTGF; **b**, Fibronectin-1), angiogenic potential (**c**, VEGF) and inflammation (**d**, IL-6) was assessed in iA-ASM cells using qRT-PCR. Data represent mean±SD (n=3). Results from cultures transfected with empty vector (■), Runx2V1$_L$ (■), Runx2 V1(■) and Runx2 V2 (■) are shown.
