# Supplementary material for: Runx2 Regulated Airway Homeostasis Is Disrupted in Asthma
Source: FASEB J. 2026 Feb 17;40(4):e71544. doi: 10.1096/fj.202502088R (PMC12911552; doi:10.1096/fj.202502088R)
Supplement: Supplementary file 5 — Figure S5: fsb271544‐sup‐0005‐FigureS5.pdf. [file FSB2-40-e71544-s002.pdf]

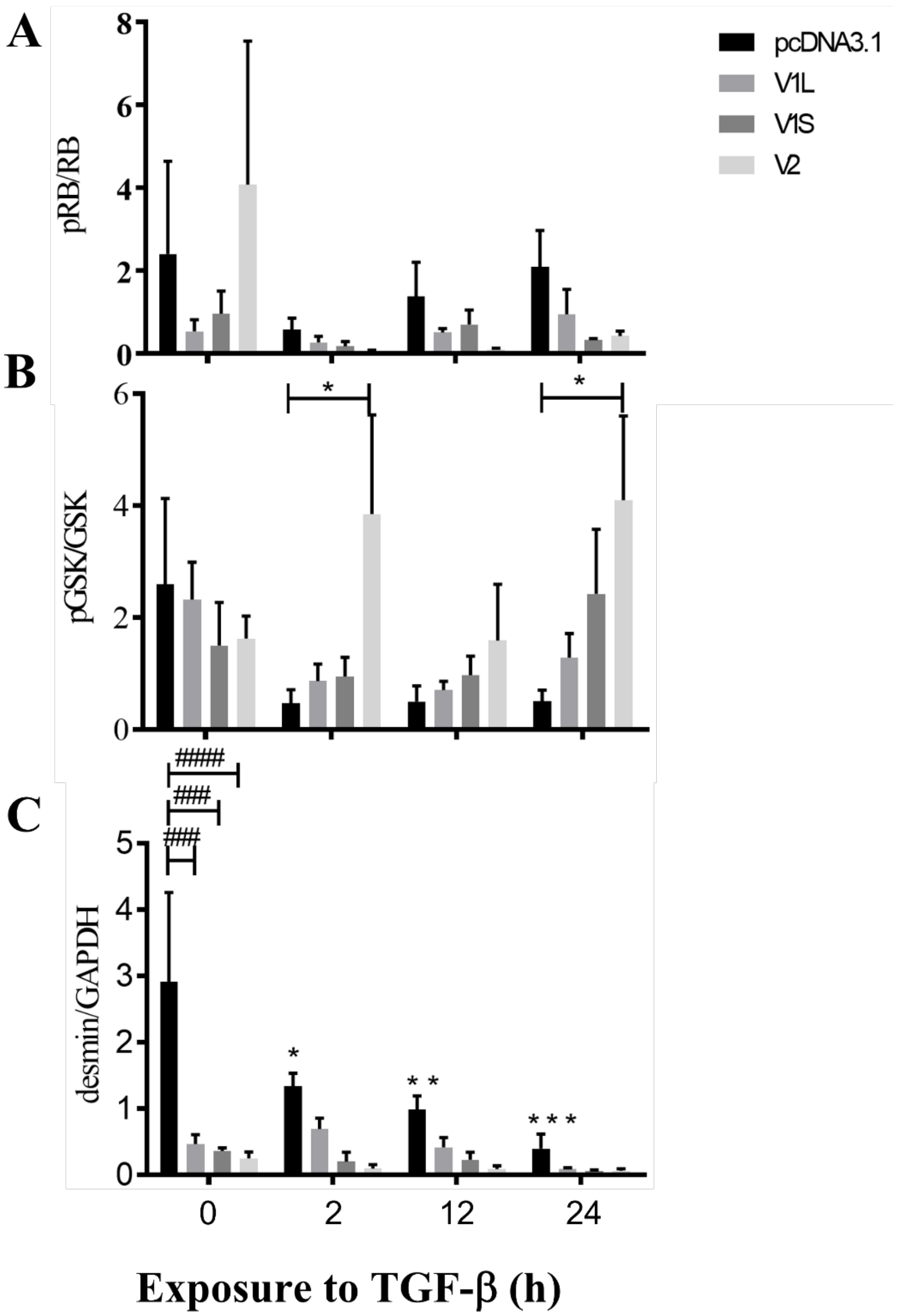

Supplementary Figure 5 Quantification of the decrease in ASM remodeling markers by Runx2 isoforms in iA-ASM cells. iA-ASM cells were transfected with empty vector (■), Runx2 V1L (■), Runx2 V1(■) or Runx2 V2 (■) and stimulated with TGF-β (1ng/ml) for up to 24 h. Changes in expression of markers of proliferation (**a,** p-Ser^{795}/total pRB) and hypertrophy (**b,** p-Ser9/total GSK 3β; **c,** desmin) on immunoblot images (fig. 7) were quantified using image J software (n=3). *p<0.05, **p<0.01, ***p<0.001 denotes significance between BSA and TGF-β. ###p<0.005, #### p≤0.001, #####p<0.0001 # indicates significant difference between NA- and A-ASM cells.
