# Supplementary material for: Runx2 Regulated Airway Homeostasis Is Disrupted in Asthma
Source: FASEB J. 2026 Feb 17;40(4):e71544. doi: 10.1096/fj.202502088R (PMC12911552; doi:10.1096/fj.202502088R)
Supplement: Supplementary file 6 — Table S1: fsb271544‐sup‐0006‐TableS1.pdf. [file FSB2-40-e71544-s006.pdf]

Supplementary Table 1. Predicted analysis of the transcription factor binding elements in the human CTGF promoter (-5400 to +200 relative to transcriptional start site)

Transcription Factor	Distance from translational start site (bp)
AABS	-5091, -1769, -1485
Ad2MLP	-4916, -4558, -4419, -4024, -3463, -1560, -1339, -725, -706, -306, -30
αγSTAT	-4621, -767
**αCE2Maf**	**-5372, -4829, -4757**
α-INF.2(6)	-5450, -5409, -5184, -4834, -4693, -4666, -4531, -4035, -3933, -3726, -2644, -2217, -1428, -1396, -1284, -604, -597, -378, -301
**ANF**	**-5181, -4363, -4186, -4041**
AP-1	-4832, -4760, -4676, -4016, -3253, -3197, -3072, -2767, -2766, -2696, -1807, -1420, -1053, -653, -293
AP2	-5418, -5395, -5308, -5206, -5205, -5204, -5063, -4878, -4806, -4726, -4683, -4484, -4466, -4465, -4464, -4421, -4299, -4263, -4208, -4054, -4049, -3703, -3456, -3025, -453, -15, 68, 71, 129, 145, 164
AP-2α/γ	-5418, -5391, -5334, -5310, -5291, -5238, -5203, -4885, -4868, -4796, -4684, -4571, -4568, -4498, -4465, -4180, -4134, -4078, -3961, -2221, -2119, -1791, -1758, -1132, -40, -23, -23, 49, 76, 137, 186
AP-3	-4911, -4897, -4888, -4816, -3841, -3659, -1958, -955
AP-4	-4325, -2940, -605
BRE	-5287, -5210, -5189, -4306, -4283, -4174, -4057, -4025, -1739, 170
Brn-2	-3073, -2995, -2767, -1088
C/EBP	-5415, -5090, -4912, -4895, -4889, -4817, -4524, -3607, -3561, -2813, -1806, -1384, -1113
C/EBP-p62	-1901, -1806
c-Abl	-3742, 4
CAC	-4892, -3542, -674, -110, 114
CARG	-383
CBFA1-CE1	-655
Cdx	-4916, -4660, -4024, -3474, -3463, -3308, -2128, -1344, -304
**c-fos**	**-4704**, -2232
**CHX10**	**-4836, -4764, -4280**, -2712
c-mos	-3842, -2568, -967, -933
c-Myb	-5321, -4905, -4899, -4795, -4612, -4494, -4393, -4283, -3520, -3068, -2523, -1763, -616
c-Myc	-4106, -705
**Cited-2**	**-4978**
**CNBP-SRE**	**-4415, -4172, -3960**
CP1/MLP	-2717, -2709
CP2-γ	-2402, -1307
CR	-5193, -5156, -5069, -4983, -4966, -4550, -3491, -3452, -3074, -2939, -2783, -2698, -2342, -2196, -2055, -1578, -1475, -1094, -941, -494, -436
CR2	-4552, -3073, -2348, -1510

CRE	-2962
**CREB**	**-5259, -4492**
CTF	-5187, -4959, -3355, -2689, -957, -59
DRE	-2101, -836, -157
E1A	-5333, -4469, -4382, -4038, -3661, -1922, -387, -375
E2A	-5321, -5280, -4374, -4323, -4192, -3343, -1188
E2F/DP	-5318, -5316, -5210, -4415, -4349, -4305, -4174, -4056, -4024, -248, -8, 101, 171
E47	-5300, -4822, -3947
E4BP4	-3560, -1342
E4F1	-5259, -2961
Ear2	-4959, -4156, -1597, -1142
Early-SEQ1	-4743, -4731, -4722, -4710, -4700, -4689, -2062, 182
**EBP1**	**-4887, -4815**
**EBV/ZRE**	**-4902, -3606**, -1995
**ECI-6/IκB-α**	**-4885, -4813**, -2950
EF-1A	-5299, -4382, -4038, -378
**EGR-1**	**-4263**
**EGR-2**	**-4263**
EGR-1/Krox-24	-4300, -4264, 164
ELK-1	-2777, -2198, -379, 15
**ESE-1**	**-5407, -5300, -4383, -4359, -4070**, -3204, -2343, -2199, -1660
ESE-2	-5108, -4667, -4535, -4146, -2020, -1660, -222
**ESE-3**	**-5299**
Ets-1	-5299, -4358, -2198, -659, -118
Forkhead	-5375, -5135, -5126, -5075, -5020, -4960, -4416, -4257, -4228, -4128, -4064, -5126, -5075, -5020, -4931, -4661, -3807, -3778, -3556, -3179, -3134, -2897, -2783, -2597, -2563, -1829, -1478, -1439, -1204, -408, -308, -83
FOX Family	-4931, -4661, -3807, -3778, -3556, -3179, -3134, -2897, -2783, -2597, -2563, -1829, -1478, -1439, -1204, -956, -408, -308, -83
FoxO	-5140, -3753, -3745, -3555, -3536, -3229, -3117, -1317, -1301, -976, -883, -308
**FRE**	**-5350, -5060**
FREAC	-4661, -4128, -3754, -3556, -308, 23
GATA	-4981, -3868, -3599, -3589, -2936, -2780, -2771, -1837, -1575, -1472, -1464, -1219, -298
GATA-1	-5137, -5066, -4982, -4953, -4891, -4353, -3868, -3599, -3420, -2936, -2780, -2502, -2342, -2260, -2197, -2133, -1839, -1837, -1603, -1575, -1518, -1517, -1465, -1180, -789
GATA-6	-5137, -4981, -4312, -4110, -3868, -3599, -3499, -3449, -2936, -2780, -2725, -2610, -2384, -1837, -1575, -1464, -942
γ-IRE	-5259, -5236, -4934, -4181, -4145, -4035, -3937, -3810, -3781, -3203, -3140, -3032, -2860, -2698, -2634, -2555, -2495, -2457, -2359, -2256, -

	2153, -2019, -1741, -1624, -1460, -1432, -1344, -1326, -1168, -1111, -1030, -765, -668, -659, -609, -495, -417, -242, -238, -157
GC-Box	-5206, -5203, -4744, -4732, -4723, -4721, -4711, -4701, -4263, -4237, -3962, -2313, -2063, -448, -12, 146
GCF	-5315, -5313, -5209, -5185, -5158, -4741, -4720, -4687, -4442, -4304, -4260, -4240, -4203, -4173, -4165, -4057, -4025, -4021, -3961, -3895, -3155, -2063, -1759, -49, -18, -11, 170, 147, 99, 70
Genesis	-3724, -2595, -263
GG-II/GG-I	-5403, -5107, -4968, -2913, -680, -240
GKLF	-5254, -5209, -5176, -5080, -4892, -4746, -4489, -4451, -4326, -4050, -4023, -3966, -3701, -3540, -2335, 172
GR/PR	-4122, -2489, -1986, -785, -636, -357
GRE	-2491
H-2RIIBP/T3R-α	-4030, -1141
H4TF-1	-2947, -679, -543
H-APF-1	-3005, -765, -242
HBP1/N-myc	-4695, -1336
**HC3**	**-4114**, -2462
**HCR**	**-3575**, -1878
**HIF-1α**	**-5262, -5332, -4494, -4483**
HNF-1	-2715
HNF-3	-5075, -4963, -4945, -4938, -2897, -2597, -2563, -1624, -941, -801, -408, -307, -145, -83
HNF-4	-5254, -1363
HNF-5	-5126, -5075, -5020, -4931, -3807, -3778, -3134, -2597, -2563, -1829, -1478, -408, -83
HNF-6	-4981, -4940, -4555, -3788, -1623, -1579
Hox-1	-4837, -4765, -2392, -1879, 34
Hsp70	-5203, -4742, -4422, -4044, -3962, -2689, -2050, -1747, -1734, -957, -681, -12, 146
**IA-1**	**-3922**, -2151
IBP-1	-5450, -5409, -4035, -2941, -604
ICP4	-2352
IE1.2	-5186, -5094, -5037, -4885, -4813, -4182, -3003, -1982, -1154, 105
IRF-1	-405
IRF-2	-5450, -5024, -4533, -2752, -1874, -1284, -704, -335, -220
IRS	-4945, -1612
Isl-1	-5397, -4947, -4835, -4763, -4281, -3987, -2712, -1642, -1245, -457
junB	-1203
**KBF-1**	**-4575**
KLF15	-4689, -4260, -3704, -2063, -448
**KROX24**	**-4263**
LF-A1	-5294, -5013, -4633, -4379, -4287, -3935, -3711, -3637, -2715, -1458, -1362, -865, -149

**LOB**	**-4673**
LSF/SV40	-5157, -5040, -4741, -4729, -4720, -4716, -4708, -4698, -4687, -4259, -3961, -3894, -2062, -42, -15, -11, 68, 184
Lva-Mo-MuLV	-4963, -3202, -2490, -2175, -1593, -356
LyF/Ikaros	-4569, -3516, -2914, -2144, -418, -266
**MAF**	**-4889, -4382**
MAZ	-5163, -4745, -4027, -3900, -746, -106, 148
MBF-1	-5286, -5134, -2302, -1184
MCM-1	-4964, -4551, -2341, -383
MED-1	-5395, -4872, -4800, -4718, -4571, -4565, -4523, -4081, -2157, -2059, -1788, -37, 152, 181
MEF-2	-4946, -4790, -2928, -1613, -981
MEF-3	-3973, -145
MIZ-1a/b/c	-5398, -5204, -5183, -5082, -4871, -4850, -4665, -4605, -4532, -4380, -4158, -3708, -2068, -1967, -1427, -1152, -1140, -1117, -836, -609, -571, -219
MOVO	-5308, -5206, -4423, -4263, -4020, -3958, -2312, -2065, -1075, -448, -109, 16, 114, 145, 155
MRE	-5318, -5286, -5079, -3929, -2508, -2302, -2167, -1184, -916, -858
Msx1	-3735, -3129, -320
**Msx2**	**-3986**
MtBF	-5401, -5066, -4571, -3494, -3311, -2761, -2502, -2260, -2133, -1425, -1291
**MyoD/MCK**	**-5300, -5007, -4820, -3947, -3434**, -3192, -3115
NF-1	-2020, -56
NFATc3	-5404, -5095, -5047, -4969, -4870, -4814, -4767, -4575, -4535, -4347, -4067, -3004, -2050, -1983, -1155, -764, -681, -241, -222
NF-E1	-4982, -4953, -4447, -4354, -3869, -3864, -3600, -2690, -2383, -2338, -2261, -2131, -1603, -1574, -1519, -1465, -1220, -1096, -943, -789
NF-E2	-4833, -4761, -656, -286
**NF-E4**	**-5259**
NFκB	-5403, -4885, -4813, -4574, -4442, -2144, -1427, -1073, -92, -56
NF-Y	-5194, -5089, -5024, -4077, -4964, -4640, -4261, -3760, -3440, -3249, -3033, -2394, -2057, -1208, -960, -488, -369, -143, -62
Nkx-2.5	-2940, -2458, -1304, -1255, -1066, -606, -319
Nkx-3.2	-5451, -5410, -4915, -4696, -3051, -2957, -2940, -1350, -914, -605, -160
Oct-1	-4931, -4853, -4781, -1204, -998, -467
**Oct-3**	**-4660**, -3308, -2910, -2764
Octa-1	-4852, -4780, -857
**Octa-2**	**-4843, -4771**
OctT3	-3775, -2936, -2128, -307
OVO	-3356
p300	-1136
p53	-3013

**Pax6**	**-5292, -5286**
pdx-1	-2713
PEA1	-4832, -4760, -4675, -653
**PEA2**	**-5326**
PEA3	-5404, -5299, -5182, -5093, -5047, -4870, -4853, -4767, -4533, -4381, -4345, -4181, -4168, -4067, -4039, -2198, -1981, -1658, -1153, -387, -220, -115
**PEBP2**	**-4395, -3953,** -2327
Pet-1	-5300, -4535, -3003, -1660, -379, -336, -222
**Pit-1**	**-4555,** -3073, -2767
PPAR	-4959, -4156, -1597, -1142
PTF1	-4086, -3408, -1333
Pu Box	-5405, -5048, -4854, -4802, -4356, -4068, -4070, -169, -104, -95
PuF	-840, -453
RORα	-4959, -4417, -4379, -4351, -4150, -3935, -3716, -3302, -1597, -1142, -646, -149, -89
**RUNX**	**-5415, -5326, -5016, -4152, -3840**
RVR	-2933
SAP-1	-2198
SIF	-2659
Smad	-4300, -4290, -3983, -174, -164
SOX	-5011, -4536, -3858, -2647, -1546, -785, -758, -639
Sox9	-785
Sox17	-785
Sox18	-325
Sp1	-5335, -5285, -5244, -5203, -5157, -5117, -5061, -5041, -5017, -4820, -4745, -4742, -4732, -4729, -4723, -4721, -4711, -4711, -4708, -4701, -4698, -4690, -4687, -4641, -4443, -4422, -4419, -4415, -4265, -4261, -4067, -4056, -4044, -4024, -4012, -3965, -3962, -3960, -3894, -3839, -3703, -3694, -2684, -2437, -2221, -2208, -2066, -2063, -1752, -1747, -1743, -1737, -1734, -1630, -1481, -840, -744, -110, -108, -48, -42, -15, -12, 43, 64, 68, 81, 101, 115, 119, 143, 146, 167, 169, 171, 181, 184
Spi/PU.i	-5408, -5151, -5035, -4997, -4969, -4805, -4670, -4552, -4399, -4299, -4288, -4139, -4042, -3786, -3755, -3691, -3684, -2813, -2768, -2733, -2241, -2052, -1900, -1672, -1565, -1430, -1335, -1255, -1232, -1224, -926, -778, -705, -531, -388, -380, -337, -330, -230, -222
SREBP1	4620, -4415 ,-4172, -3960, -2941, -2750, -1399, -1398, -606, -
SRF	-383
SRY	-786, -639
STAT1	-418, -384
STAT4	-3238, -3194
**SV40**	**-4905, -4890, -4886, -4876, -4856, -4838, -4836, -4818, -4814, -4804, -4784, -4766, -4622**, -3004
T3R	-1326, 112
TATA box	-4997, -4916, -4729, -4558, -4419, -4024, -3901, -3474, -3463, -1560, -

	1342, -1341, -1339, -926, -727, -725, -473, -304, -29
Tbx2	112
Tbx5	-5302, -5198, -5182, -5010, -4893, -4821, -4327, -4261, -4256, -4051, -3967, -3949, -3702, -3540, -3433, -833, -249
TCF/Lef1	-5373, -5015, -5011, -4907, -4560, -4536, -4503, -4359, -4195, -3751, -3743, -3740, -3545, -3522, -3413, -3386, -3289, -3178, -3058, -3016, -3012, -2938, -2901, -2705, -2647, -2553, -2455, -1831, -1546, -1460, -1439, -1299, -1193, -1021, -1002, -974, -639, -493, -126, 6
βTCR	-3547, -2900, -1377
TEF-1	-5078, -4909, -4892, -4856, -4847, -4784, -4775, -4727, -3831, -3223, -2431, -1220, -853, -377, -168, -103, -94
TFIID	-5011, -4461, -4143, -4098, -4013, -3776, -3665, -3132, -2796, -1779, -1666, -1545, -1541, -1375, -1350, -947, -540, -69
TGFβ Responsive Element	-156
TonEBP	-4886, -4814, -4601, -4575, -2050, -681
Tst-1	-4916, -4583, -4109, -4101, -4018, -3626, -3496, -3475, -3046, -2780, -1055
UPE	-4952, -4464, -4241, -4177, -4033, -3793, -3617, -3267, -3030, -2814, -2078, -1385, -1109, -999
v-MCS	-5321, -4897, -4612, -4793, -4393, -3520, -3068, -2523, -1763, -614
**v-Myb**	**-4897, -4793, -4612,** -2523
WAP-US6	-5142, -5110, -4208, -3685, -881
W-element	-5080, -5030, -4801, -4633, -3713, -3151, -2842, -2755, -2020, -1015, -441
**X-MyT1**	**-5444, -4539**, -2989
**XRE**	**-5344**, -2250, -2228
YY1	-5384, -5257, -4553, -4490, -4080, -3873, -3728, -3584, -3529, -3487, -3447, -3030, -2817, -2770, -1226, -1200, -297
Z-box	-4987, -4851, -4779, -3608, -1570, -1487, -857, -462
**ZBED6**	**-3456**
**Zif-1**	**-4263**

Bold type indicates transcription factor binding sites contained in the upstream regulatory region (-5400 to -3400) that are not contained in the core promoter (-1500 to +200)
