# Supplementary material for: Runx2 Regulated Airway Homeostasis Is Disrupted in Asthma
Source: FASEB J. 2026 Feb 17;40(4):e71544. doi: 10.1096/fj.202502088R (PMC12911552; doi:10.1096/fj.202502088R)
Supplement: Supplementary file 7 — Table S2: fsb271544‐sup‐0007‐TableS2.pdf. [file FSB2-40-e71544-s004.pdf]

Supplementary Table 2. Expression of RUNX2 binding partner in NA- and A-ASM cells

Gene	A vs NA TGFB				
	adj.P.Val	P.Value	t	B	logFC
FOS	0.975	0.389	0.918	-4.640	0.227
ALYREF	0.985	0.780	-0.290	-4.750	-0.033
BMPR1A	0.975	0.204	1.400	-4.500	0.185
CBFB	0.975	0.192	1.444	-4.490	0.243
CREBBP	0.975	0.335	1.036	-4.610	0.147
EP300	0.987	0.807	0.254	-4.760	0.042
ETS1	0.984	0.753	-0.328	-4.750	-0.156
FBXW12	0.977	0.536	0.650	-4.700	0.110
FBXW7	0.987	0.802	-0.260	-4.760	-0.045
HDAC1	0.984	0.756	-0.323	-4.750	-0.094
HDAC3	0.975	0.410	0.877	-4.650	0.115
HDAC4	0.984	0.700	0.402	-4.740	0.065
HDAC5	0.991	0.864	0.178	-4.760	0.031
HDAC6	0.975	0.379	0.940	-4.630	0.116
HDAC7	0.983	0.654	0.468	-4.730	0.078
HIF1A	0.977	0.521	-0.676	-4.700	-0.234
JUN	0.977	0.518	-0.680	-4.690	-0.187
KAT2B	0.985	0.769	-0.306	-4.750	-0.061
KAT6B	0.975	0.488	-0.732	-4.680	-0.098
NEDD1	0.984	0.745	-0.338	-4.750	-0.041
NR0B2	0.975	0.440	-0.819	-4.660	-0.137
PML	0.975	0.483	0.741	-4.680	0.131
RB1	0.975	0.237	-1.294	-4.540	-0.270
RBM14	0.975	0.413	-0.870	-4.650	-0.185
SKP2	0.979	0.572	-0.593	-4.710	-0.094
SMAD5	0.981	0.606	0.540	-4.720	0.126
SMURF1	0.975	0.246	1.265	-4.540	0.139
SMURF2	0.991	0.859	0.184	-4.760	0.043
SOX9	0.975	0.157	1.586	-4.450	0.587
STAT3	0.975	0.324	1.061	-4.600	0.264
STAT5A	0.975	0.420	0.858	-4.660	0.248

STUB1	0.975	0.410	-0.876	-4.650	-0.115
SUMO1	0.984	0.699	0.403	-4.740	0.054
TLE1	0.975	0.223	-1.338	-4.520	-0.349
TP53	0.975	0.348	1.007	-4.620	0.138
**UBTF**	**0.975**	**0.041**	**2.510**	**-4.170**	**0.373**
**WWP1**	**0.975**	**0.029**	**2.737**	**-4.110**	**0.425**
XRCC5	0.984	0.731	0.358	-4.750	0.069
YAP1	0.981	0.601	-0.547	-4.720	-0.111

Bold text represents significantly changed abundance.
